# Single-cell multi-omics sequencing of mouse early embryos and embryonic stem cells

**DOI:** 10.1038/cr.2017.82

**Published:** 2017-06-16

**Authors:** Fan Guo, Lin Li, Jingyun Li, Xinglong Wu, Boqiang Hu, Ping Zhu, Lu Wen, Fuchou Tang

**Affiliations:** 1 Beijing Advanced Innovation Center for Genomics, Ministry of Education Key Laboratory of Cell Proliferation and Differentiation, College of Life Sciences, Peking University, Beijing 100871, China;; 2 Biomedical Institute for Pioneering Investigation via Convergence, Peking University, Beijing 100871, China;; 3 Peking-Tsinghua Center for Life Sciences, Peking University, Beijing 100871, China;; 4 Group of Translational Medicine, Department of Obstetrics and Gynecology, Ministry of Education Key Laboratory of Obstetric, Gynecologic & Pediatric Diseases and Birth Defects, West China Second University Hospital, Sichuan University, Chengdu, Sichuan 610041, China;; 5 Academy for Advanced Interdisciplinary Studies, Peking University, Beijing 100871, China

**Keywords:** epigenome, single-cell COOL-seq, multi-omics sequencing, mouse preimplantation embryos, reprogramming

## Abstract

Single-cell epigenome sequencing techniques have recently been developed. However, the combination of different layers of epigenome sequencing in an individual cell has not yet been achieved. Here, we developed a single-cell multi-omics sequencing technology (single-cell COOL-seq) that can analyze the chromatin state/nucleosome positioning, DNA methylation, copy number variation and ploidy simultaneously from the same individual mammalian cell. We used this method to analyze the reprogramming of the chromatin state and DNA methylation in mouse preimplantation embryos. We found that within < 12 h of fertilization, each individual cell undergoes global genome demethylation together with the rapid and global reprogramming of both maternal and paternal genomes to a highly opened chromatin state. This was followed by decreased openness after the late zygote stage. Furthermore, from the late zygote to the 4-cell stage, the residual DNA methylation is preferentially preserved on intergenic regions of the paternal alleles and intragenic regions of maternal alleles in each individual blastomere. However, chromatin accessibility is similar between paternal and maternal alleles in each individual cell from the late zygote to the blastocyst stage. The binding motifs of several pluripotency regulators are enriched at distal nucleosome depleted regions from as early as the 2-cell stage. This indicates that the *cis*-regulatory elements of such target genes have been primed to an open state from the 2-cell stage onward, long before pluripotency is eventually established in the ICM of the blastocyst. Genes may be classified into homogeneously open, homogeneously closed and divergent states based on the chromatin accessibility of their promoter regions among individual cells. This can be traced to step-wise transitions during preimplantation development. Our study offers the first single-cell and parental allele-specific analysis of the genome-scale chromatin state and DNA methylation dynamics at single-base resolution in early mouse embryos and provides new insights into the heterogeneous yet highly ordered features of epigenomic reprogramming during this process.

## Introduction

Single-cell sequencing technologies have greatly facilitated the dissection of the heterogeneity of populations of cells^1,2,3,4,5,6,7,8,9^. Recently, we and others have developed single-cell epigenome sequencing technologies that include single-cell DNA methylome sequencing (scRRBS and scBS), single-cell Hi-C, single-cell ChIP-seq, single-cell DamID, single-cell DNaseI-seq and single-cell ATAC-seq, to dissect the epigenetic heterogeneity of cell populations^10,11,12,13,14,15,16,17,18^. Moreover, we and other groups have developed single-cell DR-seq, single-cell M&T-seq and single-cell Trio-seq methods that can simultaneously analyze genome-transcriptome, DNA methylome-transcriptome and genome-transcriptome-DNA methylome data from individual cells^19,20,21^. However, the simultaneous measurement of all the different layers of epigenomic information from the same individual cell has not yet been achieved. Here, we report the development of a single-cell multi-omics sequencing technology called single-cell COOL-seq (Chromatin Overall Omic-scale Landscape Sequencing), which can simultaneously analyze the chromatin state, nucleosome positioning, DNA methylation, copy number variation (CNVs) and ploidy from the same single cell.

During mouse preimplantation development, a global epigenetic event reprogrammes the highly differentiated gametes to totipotent embryos. This is highlighted by global DNA demethylation^22,23,24,25,26,27,28,29,30,31,32,33,34,35,36,37,38^. However, the epigenomic heterogeneity between individual cells of mouse preimplantation embryos has never been addressed. Here, we used the single-cell COOL-seq technique to analyze mouse preimplantation embryos at seven consecutive developmental stages (early zygotes when the male and female pronuclei are still separated from each other, late zygotes, 2-cell embryos, 4-cell embryos, 8-cell embryos, morulae and blastocysts) (Supplementary information, Table S1).

## Results

### Development of single-cell COOL-seq technology

**Figure 1:**
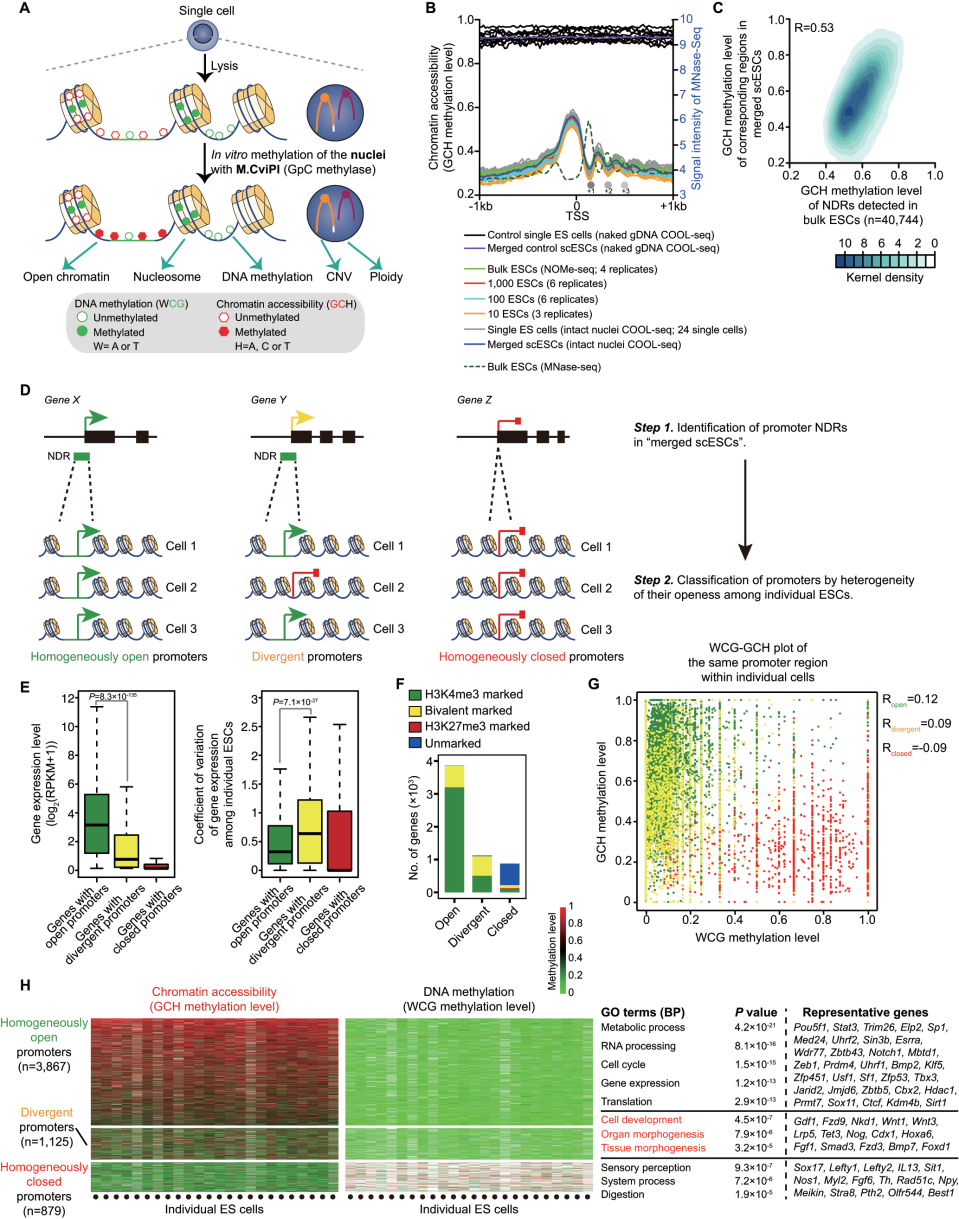
Establishment of single-cell COOL-seq in mouse embryonic stem cells. **(A)** Diagram of the single-cell COOL-seq method. **(B)** Chromatin accessibility of individual mouse ES cells around the transcription start site (TSS) revealed by single-cell COOL-seq. Average GCH methylation levels, which reflect the chromatin openness of bulk (marked with green), titration series (from 1 000 cells to 10 cells) or single ES cells (marked with gray), are marked with solid lines. The dashed curve represents the signal intensity of the nucleosome positioning in bulk mouse ES cells from published MNase-seq data. As a control, we also detected *in vitro* DNA methylation of naked genomic DNA of individual ES cells (marked with black). Note that the solid circles (+1, +2 and +3) represent the first three common strongly positioned nucleosomes downstream of the TSS identified by both scCOOL-seq and bulk cell MNase-seq. **(C)** Correlation of global chromatin accessibility profiles between scCOOL-seq and bulk NOMe-seq data. A total number of 40 744 of NDRs found in the bulk NOMe-seq data was used, these regions were detected in our merged scCOOL-seq containing at least five GCH sites, which were ≥ 5× sequencing depth. **(D)** Classification of genes promoters into homogeneously open, homogeneously closed and divergent groups. 9 685 promoter NDRs identified in merged ES cells were used. **(E)** Gene expression and coefficient of variation of the corresponding genes with homogeneously open promoters, homogeneously closed promoters and divergent promoters among individual ES cells. **(F)** The number of genes within each category that had either H3K4me3 or H3K27me3 marks in mouse ES cells was calculated. **(G)** Dot plot of WCG methylation (endogenous DNA methylation) and GCH methylation (chromatin accessibility) level of the same promoter regions within individual cells. **(H)** Chromatin accessibility and DNA methylation level of homogeneously open promoters, homogeneously closed promoters and divergent promoters of individual ES cells. Right panel represents the GO terms for each group of genes.

A powerful epigenome sequencing technology known as NOMe-seq (Nucleosome Occupancy and Methylome Sequencing) was developed several years ago^39,40,41,42,43,44^. However, it requires hundreds of thousands of cells as starting material. Here we have combined NOMe-seq and PBAT-seq (Post-Bisulfite Adaptor Tagging Sequencing) methods and systematically modified them by serial titration assays to improve the sensitivity to provide robust output at single-cell resolution (Figure 1A and 1B, Supplementary information, Figure S1 and Data S1). We are also able to spike in the same quantity of lambda DNA into each single-cell sample to determine the ploidy of the cell. In total, we sequenced 24 single mouse embryonic stem (ES) cells and pooled counterpart cells (bulk cells). As a control, we also performed *in vitro* DNA methylation of naked genomic DNA of individual ES cells (Figure 1B).

We could show that the single-cell COOL-seq technique provides highly digitized data on DNA methylation at single-base resolution (Supplementary information, Figure S2A and S2B). When we compared our scCOOL-seq-generated DNA methylation data of single ES cells and single oocytes arrested in meiosis II (MII oocytes) with a published DNA methylation data set of single mouse ES cells and MII oocytes generated by scBS^16^ (Supplementary information, Figure S2C), we found a robust and highly accurate detection of DNA methylation at single-base resolution by the scCOOL-seq method (Supplementary information, Figure S2C). The characteristic patterns of open chromatin and nucleosome positioning at promoter regions were also clearly detected in our scCOOL-seq analysis of 24 ES cells, but not of 10 single control ES cells with naked genomic DNA (Figure 1B; marked with black). Using scCOOL-seq, we could detect 67 168 nucleosome depleted regions (NDRs) from the aggregated scCOOL-seq data of all 24 ES cells (Supplementary information, Table S1). Of these 67 168 NDRs, 36 071 were consistent with DNaseI-seq data from the bulk ES cells (Supplementary information, Figure S2D). The NDRs identified only in the scCOOL-seq data set but not in the DNaseI-seq data set still displayed a clear enrichment for H3K4me1 and H3K4me3 (Supplementary information, Figure S2E). They also showed clear depletion of nucleosomes, as indicated by MNase-seq analysis (Supplementary information, Figure S2E). Although the average number of GCH sites covered in an individual cell is 10% of that of the bulk cells under current sequencing depth (∼2× (6.0 Gb) coverage), more GCH sites can be recovered by merging the single-cell data together whereupon the characteristic patterns of chromatin accessibility at both promoters and NDRs can be faithfully reproduced in analyses of merged single ES cells (Figure 1C and Supplementary information, Figure S3). The NDRs detected in single ES cells showed significant enrichment in the CpG islands (CGIs), DNaseI-hypersensitive sites (DHS), CTCF-binding sites, H3K4me3-marked promoters and enhancers, but were depleted at repetitive elements such as LINEs, SINEs and LTRs (Supplementary information, Figures S2F, S2G and S4). Chromatin accessibility around gene promoter regions was highly associated with gene expression and CpG density by scCOOL-seq, as expected (Supplementary information, Figure S4C and S4D). By performing scCOOL-seq, we could determine the positional information for 826 524 nucleosomes from the aggregated scCOOL-seq data of all 24 ES cells (Supplementary information, Table S1). The nucleosome positions detected in single ES cells were consistent with those determined by MNase-seq analysis of bulk ES cells and the characteristic pattern of nucleosome positioning around CTCF binding sites could also be reproduced in the manner expected (Supplementary information, Figure S5). To further confirm the robust and accurate detection of both open and closed chromatin by the scCOOL-seq method, we validated the chromatin status of selected genomic loci using a low-input and locus-specific chromatin status analysis method named liDNaseI-qPCR^34^ (Supplementary information, Figure S6). Thus, our scCOOL-seq method can be readily used to determine both chromatin accessibility and nucleosome positioning.

Due to the still relatively low coverage of current epigenome sequencing technologies, the current corresponding pipeline used for identifying potential *cis*-regulatory elements in bulk cells will poorly define genomic features in single-cell sequencing data with a sparse nature. Thus, evaluation of the robustness and analysis of cellular variation across individual cells is impractical by current strategies used with bulk cells. However, this problem can be resolved by using the genomic features defined first in the aggregated (merged) single-cell data set and then by quantifying the variance among individual cells in these regions^10,11,13,15^. Considering this, we developed an updated pipeline for scCOOL-seq to robustly measure genomic features across individual cells with high accuracy (Supplementary information, Figure S7A and S7B). We found that in each individual cell, an average of 49.2% of the corresponding regions of promoter NDRs, 49.3% of the corresponding regions of proximal NDRs, 38.5% of the corresponding regions of distal NDR and 28.6% of the corresponding regions of nucleosomes defined in merged single-cell samples can be covered (Supplementary information, Figure S7C). Moreover, in each individual cell, over 70% of the covered regions corresponding to promoter NDRs defined in merged single ES cell samples were also detected as open chromatin (Supplementary information, Figure S7D). Furthermore, in each individual cell, over 80% of covered regions corresponding to nucleosomes defined in merged single ES cell samples were also detected as closed chromatin (Supplementary information, Figure S7D). These data suggested high accuracy and robust detection of chromatin status (open or closed chromatin) across individual cells by the scCOOL-seq method.

By applying the updated pipeline for scCOOL-seq, genes can be classified into three different types based on the heterogeneity of the chromatin accessibility of the gene promoter regions in each individual cell within a cell population: genes with homogeneously open promoters between individual cells; genes with homogeneously closed promoters between individual cells; and the heterogeneously open/closed mixed state genes (divergent genes) when comparing individual cells (Figure 1D) (see the Materials and Methods section for further details). We first tested this classification in mouse ES cells, and we found that the genes with homogeneously open promoters were on average more active in transcription compared to those with divergent and homogeneously closed promoters (Figure 1E). Moreover, the homogeneously open genes in general showed less variability of gene expression among different individual ES cells (Figure 1E, they showed a much lower coefficient of variation (CV) in single-cell RNA-seq analysis). The majority (82.8%) of homogeneously open genes were marked with H3K4me3, whereas 52.9% of divergent genes were marked with both H3K4me3 and H3K27me3 (Figure 1F). Furthermore, the promoter regions of divergent genes were in general depleted of endogenous DNA methylation in each individual cell, and so the chromatin accessibility of promoters of these genes did not correlate with their endogenous DNA methylation levels within each individual cell (Figure 1G). This indicates that factors other than DNA methylation are determinants of the heterogeneity of the open versus closed chromatin states of the promoter regions of these genes. GO analysis of the divergent genes in the mESCs showed that these genes were specifically enriched for GO terms such as cell development and organ/tissue morphogenesis (Figure 1H), indicating a link between the heterogeneity of promoter status and functional states of mESCs.

### DNA methylation dynamics and heterogeneity of mouse preimplantation embryos revealed by single-cell COOL-seq

**Figure 2:**
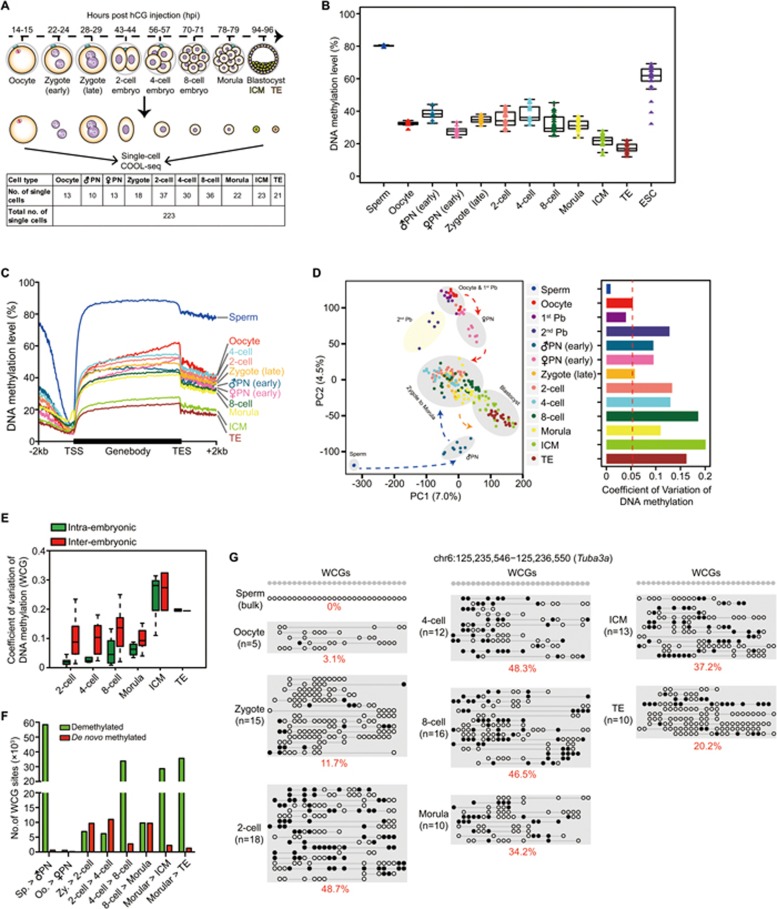
DNA methylation dynamics of mouse preimplantation embryos revealed by single-cell COOL-seq analysis. **(A)** Numbers of individual oocytes and blastomeres analyzed using the single-cell COOL-seq technique. Note that all the samples in the relevant stages used in this study were carefully examined to remove contaminated polar bodies. **(B)** Boxplot of average DNA methylation level in each single blastomere. The mean methylation level of all the detected WCG sites (≥ 2× depth) in a single cell was calculated (shown as a triangle), and cells at the same developmental stage were plotted together in the same box. The bottom and top of the boxes indicate the first and third quartiles, respectively, and the lines inside the boxes indicate the medians of the data. Note that the sperm DNA data were from bulk samples. **(C)** The average DNA methylation levels (WCG methylation level) along the gene bodies, 2 kb upstream of the TSS and 2 kb downstream of the transcription end sites (TES) of all the RefSeq genes across different developmental stages. **(D)** Principle component analysis of the DNA methylome (methylation levels of WCG sites in 5 kb windows) of the gametes, polar bodies, pronuclei and cleavage stage embryos (222 single cells and 9 bulk sperm samples). Right panel represents the coefficient of variance of DNA methylation within each stage. **(E)** Coefficient of variation (CV) of DNA methylation among single blastomeres. **(F)** Number of demethylated and *de novo* methylated WCG sites after fertilization. A WCG site with an over 0.75 methylation level at one developmental stage and with an at least 0.3 methylation level decreases at the following stage was defined as a demethylated WCG site (Benjamini-Hochberg's FDR < 0.05). A WCG site with < 0.25 methylation level at one developmental stage and with an at least 0.3 methylation level increases at the next stage was defined as a *de novo* methylated WCG site (Benjamini-Hochberg's FDR < 0.05). **(G)** Representative *de novo* methylated locus in early mouse embryos. The open white and filled black circles indicate the unmethylated and methylated WCG sites, respectively. The circles on the same line indicate the DNA methylation state in one single cell. Note that the DNA methylation state in the bulk sperm sample was the mean methylation state at each WCG sites.

Next, we used our well-defined single-cell COOL-seq technology to explore the dynamics of chromosome remodeling and epigenomic heterogeneity in mouse preimplantation embryos. In total, we analyzed 223 single cells from the oocyte stage to the blastocyst stage as well as 9 bulk samples of sperm cells (Figure 2A). The sperm DNA, which was heavily methylated compared to the oocytes, underwent dramatic DNA demethylation shortly after fertilization (80.3% median level in the sperm and 38.3% in the early male pronuclei, *P* = 1.4 × 10^−11^) (Figure 2B and 2C). The maternal genome also displayed a mild reduction in DNA methylation (32.4% in the oocyte and 27.8% in the early female pronuclei, *P* = 6.3 × 10^−5^) (Figure 2B and 2C). This phenomenon was consistent with recent studies indicating that both parental genomes undergo active and passive demethylation before the first cleavage of mouse embryos^24,25,27,30^. Moreover, the male pronuclei and female pronuclei partially retained their parental features and displayed strong heterogeneity compared to the oocytes (Figure 2D). More importantly, we found that at the 2-cell embryo stage, DNA methylation heterogeneity within an embryo is much lower than that between different embryos (Figure 2E) (*P* = 1.2 × 10^−16^), indicating that the DNA methylation reprogramming between the blastomeres within the same 2-cell embryo is highly synchronized, probably due to the highly synchronized cell cycle of the blastomeres within the same embryo.

Moreover, we calculated the variation of DNA methylation globally and at specific genomic elements such as exon, intron, promoters, CGIs, repetitive elements and NDRs among individual cells of the same developmental stage (Supplementary information, Figure S8). As controls, the regions with the highest DNA methylation variance in ES cells are at poised enhancers (H3K4me1), repetitive elements (SINEs, LINEs, LTRs and satellites) and heterochromatin (H3K9me3), wheras the regions with the lowest DNA methylation variance in ES cells are at promoters and CGIs (Supplementary information, Figure S8). These results in ES cells were in accord with recently published data of scBS of mouse ES cells^16^. Interestingly, we found that the DNA methylation levels of the proximal NDRs were less heterogeneous among individual cells than those of distal NDRs both in preimplantation embryos at the same developmental stage and in ES cells. This indicates that these presumptive distal regulatory elements may show the first sign of development-specific DNA methylation during preimplantation development (Supplementary information, Figure S8).

Previous studies have reported a major step of *de novo* DNA methylation upon implantation of mouse blastocysts^45,46^. However, in the mouse preimplantation embryos, the *de novo* DNA methylation-related proteins were clearly localized to the nucleus across the cleavage stage^23,47,48^. We found that there were a total of 33 153 unique WCG sites with *de novo* methylation during the development of preimplantation embryos under our stringent criteria (Figure 2F). Additionally, 89 CGIs were methylated *de novo* from the late zygote stage to the morula stage (Figure 2G).

### Chromatin remodeling dynamics during mouse preimplantation development

**Figure 3:**
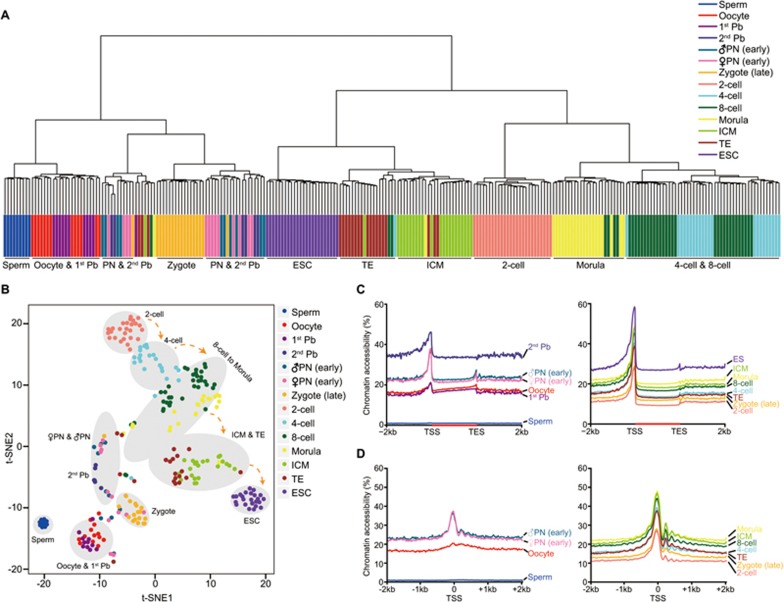
Chromatin accessibility dynamics of mouse preimplantation embryos revealed by single-cell COOL-seq analysis. **(A)** Unsupervised hierarchical clustering of mouse preimplantation embryos based on the averaged GCH methylation level of proximal NDRs (58 677 proximal NDRs) detected in all of the preimplantation stages. **(B)** t-SNE (t-Distributed Stochastic Neighbor Embedding) analysis of mouse preimplantation embryos based on the averaged GCH methylation level of proximal NDRs detected in all the stages. **(C)** The chromatin accessibility along the gene bodies, 2 kb upstream of the TSS and 2 kb downstream of the TES of all the RefSeq genes across different developmental stages. **(D)** The chromatin accessibility around TSS across all the preimplantation stages. See the legend for Figure 1B for more information.

The chromatin accessibility of promoter regions is known to be highly associated with expression levels of the corresponding genes. We therefore asked whether the chromatin accessibility of gene promoters can be used to separate different types of cells. Both unsupervised hierarchical clustering and t-SNE (t-Distributed Stochastic Neighbor Embedding) analysis clearly showed that cells at the same developmental stage were clustered together but separated from cells at other developmental stages (Figure 3A and 3B). Thus, we merged single-cell data from the same developmental stage and performed the downstream analysis. We analyzed chromatin accessibility by calculating the averaged GCH methylation level across the gene bodies and their flanking regions (Figure 3C and 3D). The gametes were less accessible around transcription start site (TSS) (TSS ± 500 bp) compared to fertilized and cleavage embryos (GCH methylation level of 1.0% in sperm, 18.0% in oocyte and 25.4% in pronucleus; oocyte versus female pronucleus, *P* = 3.5 × 10^−4^). There was also a clear increase of chromatin accessibility around TSS in the fertilized egg (Figure 3C and 3D). Moreover, the pattern of strongly positioned nucleosomes downstream of the TSS was also conserved in preimplantation embryos, ∼150 bp downstream of the TSS and 180 bp separated from each other, and was absent in the sperm and oocytes but strongly positioned after the 2-cell stage (Figure 3D). We also calculated the averaged GCH methylation level in each single blastomere and found that the most dramatic chromatin remodeling occurred in the paternal genome shortly after fertilization (sperm versus male pronucleus, *P* = 4.2 × 10^−7^) (Supplementary information, Figure S9A).

**Figure 4:**
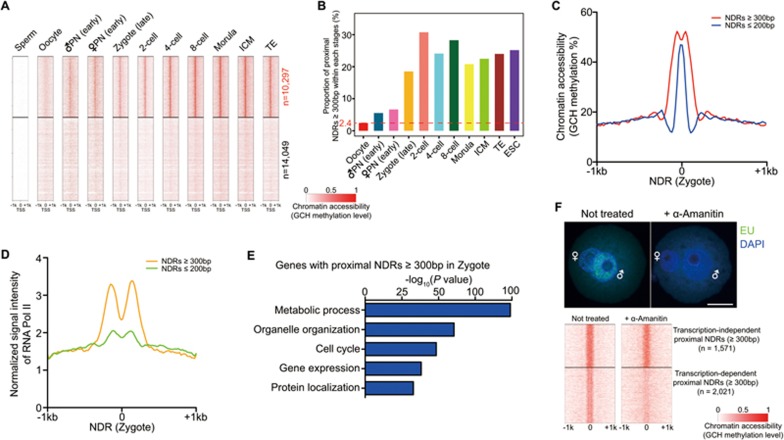
Chromatin remodeling dynamics of promoter regions during mouse preimplantation development. **(A)** Dynamics of chromatin accessibility around TSS of 24 346 RefSeq genes during mouse preimplantation development. Blastomeres within each stage were merged together and averaged GCH methylation level was calculated for this analysis. **(B)** Bar plot of the proportion of proximal NDRs with over 300 bp width during preimplantation development. **(C)** Averaged GCH methylation level around NDRs with distinct width in the zygote. **(D)** Presumptive binding of RNA Pol II around NDRs with distinct width in the zygote. Published RNA Pol II binding information of mouse ES cells by ChIP-seq was used for this analysis. **(E)** GO analysis of corresponding genes with their proximal NDRs over 300 bp in width. **(F)** Inhibition of transcriptional activity in mouse zygote and identification of transcription-dependent wider proximal NDRs after fertilization. Scale bar, 25 μm.

The open promoters detected in the blastocyst stage were gradually established after fertilization and majority of them maintained their opening throughout preimplantation development once generated (Figure 4A). We next analyzed NDRs in preimplantation embryos. Interestingly, the percentage of the wider proximal NDRs (longer than 300 bp) increased sharply from 2.4% in mature oocytes to 18.5% in late zygotes after fertilization, probably priming for zygotic genome activation at the 2-cell stage (Figure 4B and 4C). These NDRs enriched for presumptive RNA Pol II binding signals and the corresponding genes containing these wider NDRs significantly enriched for GO terms for fundamental biological processes such as metabolic processes, gene expression and cell cycle regulation (Figure 4D and 4E). To explore the effect of transcription in establishing these wider proximal NDRs within the mouse zygote, we blocked RNA Pol II-mediated transcription by its specific inhibitor, α-Amanitin. We found that inhibition of RNA Pol II activity drastically compromised the openness of these wider proximal NDRs (Figure 4F), thus indicating an important role of temporal transcription in chromatin remodeling upon fertilization.

**Figure 5:**
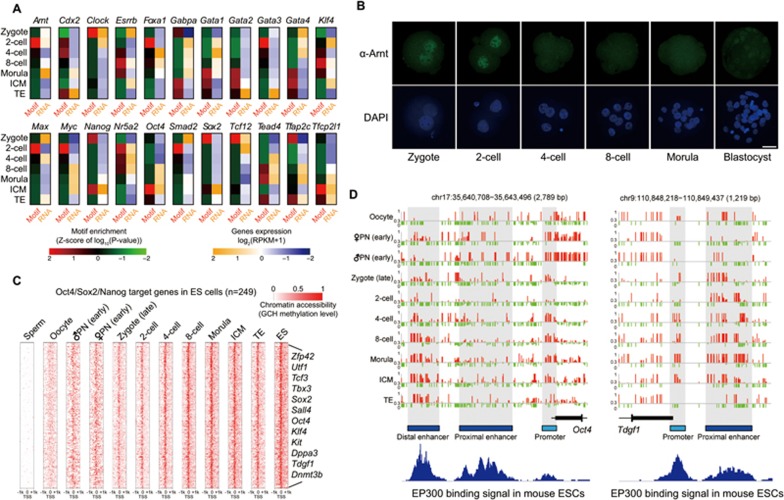
Chromatin remodeling dynamics of putative distal regulatory regions during mouse preimplantation development. **(A)** Motif enrichment analysis of distal NDRs detected in preimplantation embryos. The *z*-score of *P*-value of corresponding gene motifs and their RNA expression levels are given. **(B)** Fluorescence immunostaining of Arnt in mouse preimplantation embryos. Scale bar, 25 μm. **(C)** Dynamics of chromatin accessibility around TSS of 249 Oct4/Sox2/Nanog target genes^62^ that were open in mouse ES cells during preimplantation development. **(D)** Representative of *Oct4* gene and *Tdgf1* gene show the chromatin accessibility (GCH methylation level at single-base resolution) at their corresponding *cis* regulatory elements (promoters and enhancers) during preimplantation development.

We also asked how the open chromatin regions are potentially regulated by transcription factors. We searched for the motifs of known transcription factors in these NDRs and found that the proximal NDRs were clearly enriched for the binding motifs of the transcription factors for basic transcription machinery, such as Sp1 and E2f4 (Supplementary information, Figure S9B and Table S2). In contrast, the distal NDRs were strongly enriched for the binding motifs of a large set of master transcription factors that act in a developmental stage-specific manner (Figure 5A and Supplementary information, Table S2). For example, distal NDRs in ICM of blastocysts were clearly enriched for the binding sequence of the pluripotency master regulators Oct4, whereas those in TE were enriched for the binding motifs of trophectoderm genes *Cdx2* and *AP2γ* (Figure 5A and Supplementary information, Table S2). We also found that Arnt, whose binding motif was clearly more enriched in distal NDRs at the 2-cell stage, showed a specific localization to the nucleus at the zygote and 2-cell stages (Figure 5A and 5B). Thus Arnt may play a specific role in chromatin remodeling during mouse early embryonic development. Interestingly, the presumptive binding sites of Oct4 and EP300 have been opened from as early as the 4-cell stage, and their target genes were gradually expressed at 4-cell stage onward (Figure 5C and 5D, Supplementary information, Figure S9C and S9D), indicating that they are probably priming factors to convert the enhancer elements of pluripotency target genes into an open state long before the pluripotency is established in ICM at blastocyst stage.

Because our methods can detect both DNA methylation and chromatin accessibility simultaneously, we examined the DNA methylation levels at both NDRs and nucleosomes during preimplantation development (Supplementary information, Figure S10). When the open chromatin in gene promoters was gradually established after fertilization (Figure 4A and Supplementary information, Figure S10A), the promoters were globally hypomethylated during this whole developmental stage (Supplementary information, Figure S10A). The global chromatin accessibilities between proximal and distal NDRs were comparable within each developmental stage, while the average DNA methylation level of distal NDRs was always higher than that of proximal NDRs within each stage (Supplementary information, Figure S10B). This indicates relatively more resistance to DNA demethylation in distal NDRs compared to proximal NDRs. In contrast to ES cells that were hypermethylated in nucleosome-occupied regions, nucleosomes were kept in low DNA methylation levels throughout all of the preimplantation stages (Supplementary information, Figure S10C).

Next, we analyzed the chromatin accessibility of the functional genomic elements in the early embryos (Supplementary information, Figure S11). In general, the chromatin accessibility of different functional genomic elements mirrors that of the global patterns, yet to a variable extent. Interestingly, for the subfamilies of SINEs, the chromatin of the evolutionarily younger Alu/B1 elements was more accessible than their neighboring flanking regions at the 2-cell and 4-cell stage, whereas the chromatin of the evolutionarily older MIR elements was no more accessible than their neighboring flanking regions across the whole preimplantation development (Supplementary information, Figure S12). This points to non-synchronized chromatin remodeling in different subfamilies of SINEs.

### Tracing the dynamics of DNA methylation and chromatin accessibility of parental genomes in each individual cell

**Figure 6:**
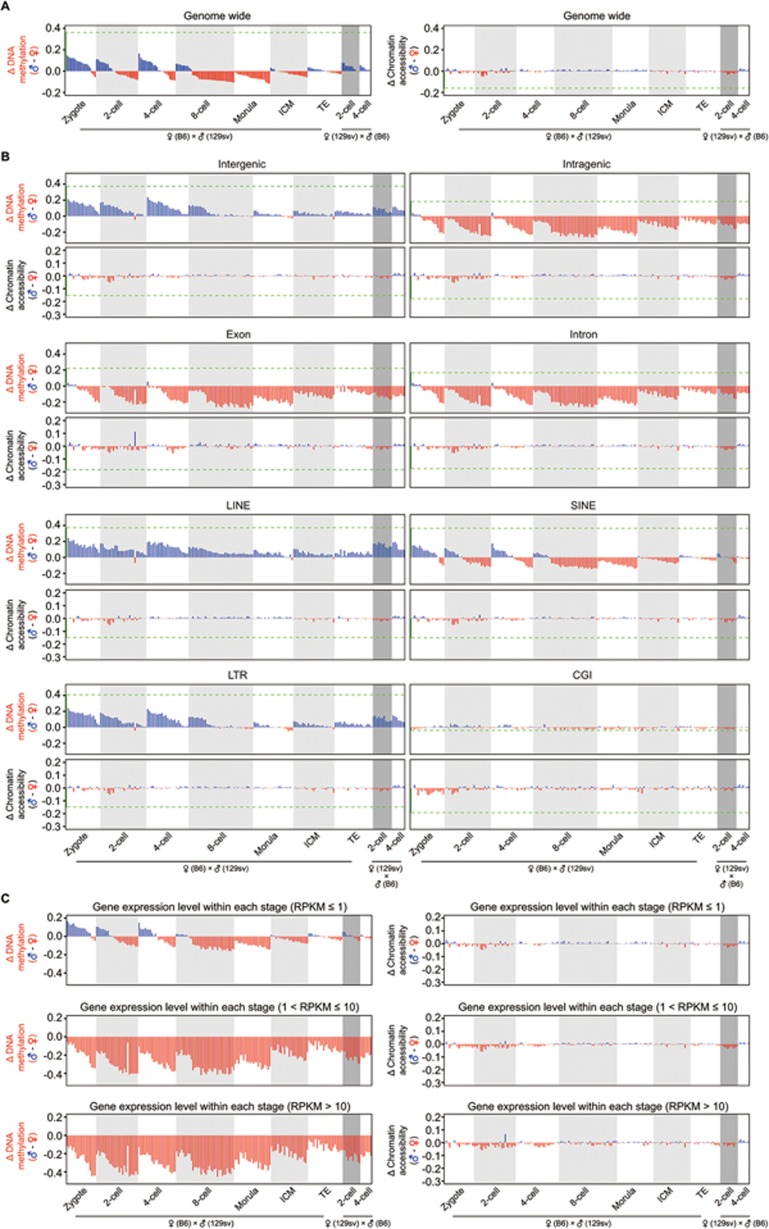
Asymmetry of DNA methylation between parental genomes within each individual blastomere during preimplantation development. **(A)** Differences of global DNA methylation level and chromatin accessibility between paternal and maternal genomes in each individual cell of mouse early embryos. The green dashed line indicates the difference of global DNA methylation level or chromatin accessibility between sperm and oocytes. The numbers of individual blastomeres from C57BL/6J female mated with 129sv male within each stage were: *n* = 18 for zygote, *n* = 26 for 2-cell, *n* = 23 for 4-cell, *n* = 36 for 8-cell, *n* = 22 for morula, *n* = 23 for ICM and *n* = 21 for TE. The numbers of blastomeres from 129sv female mated with C57BL/6J male within each stage were: *n* = 11 for 2-cell and *n* = 7 for 4-cell. **(B)** Differences of DNA methylation level and chromatin accessibility between paternal and maternal genomes at indicated genomic regions/elements in each individual cell across preimplantation development. **(C)** Differences of DNA methylation level and chromatin accessibility between paternal and maternal genomes at intragenic regions (gene body) of corresponding genes classified by their RNA expression level. Numbers of genes with their RPKM ≤ 1 analyzed in each stage were 13 547 in zygote, 13 435 in 2-cell, 13 603 in 4-cell, 13 982 in 8-cell, 13 996 in morula, 13 398 in ICM and 13 500 in TE. Numbers of genes with their 1 < RPKM ≤ 10 analyzed in each stage were 5 014 in zygote, 5 607 in 2-cell, 5 104 in 4-cell, 5 010 in 8-cell, 4 865 in morula, 5 099 in ICM and 5 029 in TE. Numbers of genes with their RPKM > 10 analyzed in each stage were 4 487 in zygote, 4 006 in 2-cell, 4 341 in 4-cell, 4 056 in 8-cell, 4 187 in morula, 4 551 in ICM and 4 519 in TE.

The sperm and oocyte that carry distinct profiles in their chromatin would undergo dramatic reprogramming upon fertilization, as shown by this study and also by others^24,25,27,30,46^. However, it remains to be determined whether there is any asymmetry of DNA methylation and chromatin accessibility between the parental genomes in each individual cell of the cleavage embryos. To explore this, we traced the maternal (C57BL/6J) and paternal (129sv) genomes in individual cells by following heterozygous SNPs between C57BL/6J and 129sv mice (see the Materials and Methods section for details). First, we confirmed a high accuracy of tracing of the parental genomes by examining the DNA methylation level of *Impact* ICR, with fully methylated maternal genome and unmethylated paternal genome across the cleavage stages, as expected (Supplementary information, Figure S13A). Next, we asked whether the parental genomes differ in their global DNA methylation level and chromatin accessibility within each individual cell. The paternal genome has a significantly higher level of global DNA methylation than maternal genome in each individual cell before the 2-cell stage (Figure 6A and Supplementary information, Figure S13B). However, the chromatin accessibility between parental genomes was comparable in each individual cell across the whole of the cleavage stages (Figure 6A; Supplementary information, Figure S13C). Moreover, the parental genomes were also comparable in their chromatin accessibility at majority of the promoters (Supplementary information, Figure S13D and S13E), indicating synchronized establishment of open chromatin in both paternal and maternal genomes after fertilization. More interestingly, when we calculated the difference in DNA methylation level between paternal and maternal genomes at different genomic regions, we found that the intergenic regions of the paternal genome showed a higher level of DNA methylation than those of the maternal genome within each individual cell from the late zygote to the 4-cell stage (Figure 6B). By contrast, the intragenic regions (including both exons and introns) of the paternal genome had a lower level of DNA methylation than those of the maternal genome within each individual cell from late zygote stage throughout preimplantation development (Figure 6B). This phenomenon of consistent asymmetry of DNA methylation level between parental genomes was also found in repetitive elements such as LINEs and LTRs, but was not observed in SINEs (Figure 6B). On the contrary, the DNA methylation level of CGIs between parental genomes was comparable in each individual cell during preimplantation development. The difference in chromatin accessibility between the parental genomes remained comparable across the whole of the cleavage stages (Figure 6B). More importantly, for the gene body regions, we found that the asymmetric enrichment of DNA methylation on the maternal alleles is much stronger for the actively transcribed genes than that for unexpressed genes (Figure 6C). We also mated female 129sv with male C57BL/6J (in contrast to female C57BL/6J with male 129sv) and traced the parental genomes in each individual 2-cell and 4-cell blastomere. The consistent asymmetry of DNA methylation between parental genomes was confirmed by their parental-specific origins, rather than allele-specific differences (Figure 6B and 6C). Together, these data indicated that the establishment of chromatin accessibility was highly synchronized between the paternal and maternal genome after fertilization, but there was strong asymmetry of DNA methylation between the parental genomes during preimplantation development, and a favorable enrichment of DNA methylation of the maternal alleles in the gene bodies was especially strong for actively transcribed genes in preimplantation blastomeres.

**Figure 7:**
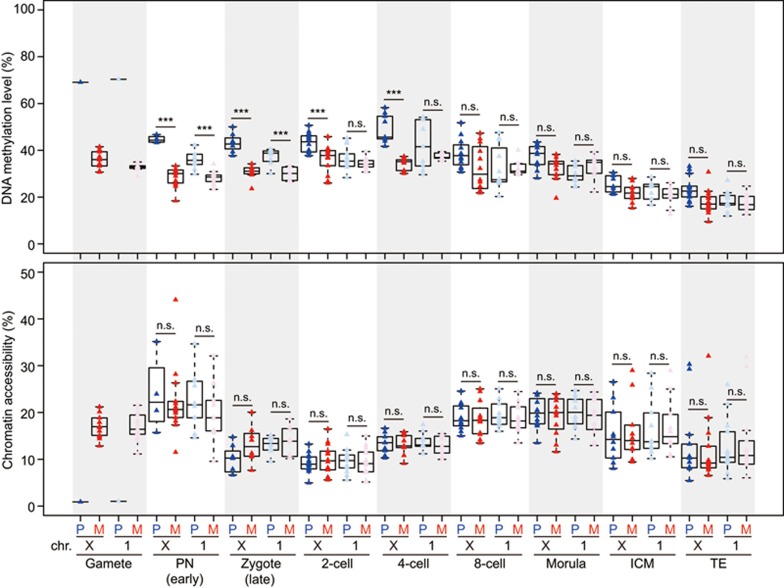
DNA methylation and chromatin accessibility dynamics of parental X chromosomes within each individual cell during preimplantation development. Dynamic DNA methylation and chromatin accessibility of paternal and maternal X chromosome within individual blastomeres from female mouse early embryos. The male and female blastomeres were distinguished by analyzing the ratio of the total reads from the X chromosome to those from chromosome 1. The mean DNA methylation levels of the X chromosome and chromosome 1 were calculated in each single cell and are shown as boxplots. A two-tailed *t*-test was used to calculate the statistical significance. ***P < 0.001. ns, not significant.

Because we can analyze the sex chromosomes of an individual cell with scCOOL-seq, we can unambiguously discriminate between male and female embryos. In this way, we analyzed the DNA methylation dynamics and chromatin accessibility of paternal and maternal X chromosomes within the same female blastomere. We found that in the male pronuclei, the DNA demethylation of the X chromosome was slower than that of the autosomes, whereas in female pronuclei, the DNA demethylation speed of the X chromosome and that of the autosomes were comparable (Figure 7). In contrast to the comparable chromatin accessibility between parental X chromosomes after fertilization, the DNA methylation level of the paternal X chromosome was significantly higher than that of maternal X chromosome from the late zygote stage to the 4-cell embryo stage (Figure 7). However, the DNA methylation level of the paternal autosomes was comparable to maternal autosomes during this same developmental period (Figure 7). When the blastocyst stage had been reached, the DNA methylation levels of both parental X chromosomes became comparable (Figure 7). And these were compatible with the fact that the paternal X chromosomes become rapidly inactivated after fertilization and reactivated in the ICM of late blastocyst stage embryos^49,50^.

### Heterogeneity of chromatin accessibility in mouse preimplantation embryos

**Figure 8:**
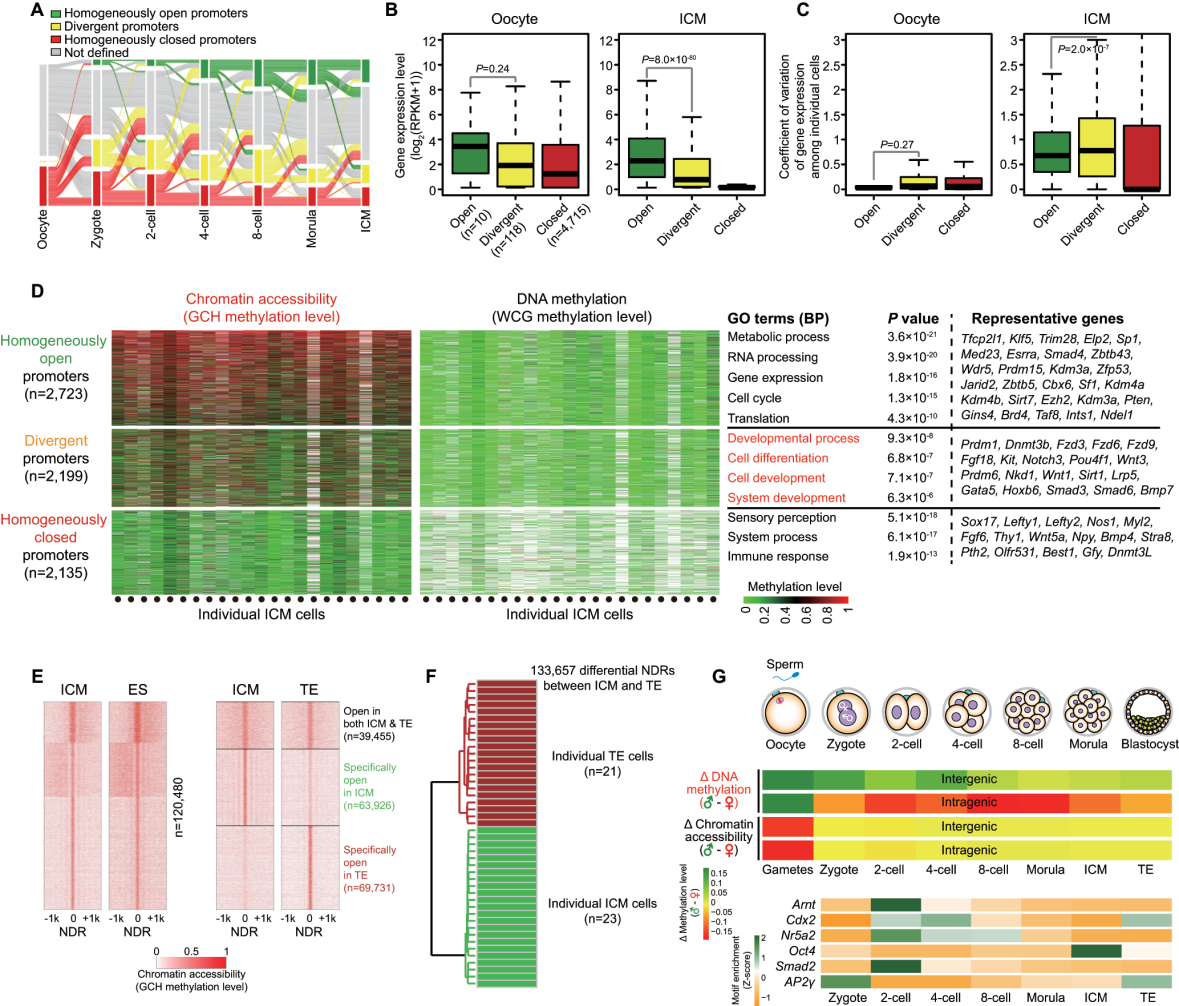
Heterogeneity of chromatin states of the promoter regions in mouse early embryos. **(A)** Alluvial plots of the dynamics of homogeneously open, homogeneously closed and divergent promoters during early embryonic development. Each line represents a gene's promoter and shows the chromatin state of genes in each stage, but lines cannot be traced for they represent different promoters during transition, and the total promoters were those classified as one of the three states in at least one analyzed stage (16 097 promoters analyzed in total). **(B)** Boxplots of RNA expression levels of genes with homogeneously open, homogeneously closed and divergent promoters in oocytes and ICM cells. **(C)** Boxplots of coefficient of variance (CV) of RNA expression levels of genes with homogeneously open, homogeneously closed and divergent promoters in oocytes and ICM cells. **(D)** Chromatin accessibility and DNA methylation level of homogeneously open promoters, homogeneously closed promoters and divergent promoters of individual ICM cells. **(E)** Comparison of global NDRs among ES cells, ICM and TE cells. NDRs from merged single cells were used for this analysis. **(F)** Unsupervised clustering of individual ICM and TE cells by differential NDRs identified between merged ICM and merged TE. **(G)** Sketch of dynamic features of DNA methylation and chromatin accessibility at single-cell and single-base resolution.

Because the NDRs within the promoter regions were highly variable among single cells within each stage during preimplantation development (Supplementary information, Figure S8), we analyzed promoter status heterogeneity in preimplantation embryos. We found that in mature oocytes, essentially all genes were homogeneously closed, compatible with the global silencing of transcription in mature oocytes (Figure 8A and Supplementary information, Table S3). After fertilization, homogeneously open genes were gradually established and reached 38.6% of the genes in the ICM (Figure 8A). However, a small proportion of genes were still kept in a homogenously closed state after fertilization. These genes were specifically enriched for GO term of regulation of T-cell activation (Figure 8A). Moreover, we analyzed the expression patterns of these categories of genes and found that throughout preimplantation development, the homogenously open genes had relatively higher levels of RNA expression and showed less variability of gene expression among individual cells, while the closed genes were not expressed (Figure 8B and 8C, Supplementary information, Figure S14). This indicates that within the first 24 h after fertilization, genes from both the maternal and paternal genomes are reprogrammed from relatively closed states to either open or divergent states and are primed for global transcription and expression during zygotic genome activation. The divergent genes in the ICM were specifically enriched for GO terms relating to developmental process and cell differentiation (Figure 8D), while these terms were observed in the homogeneous closed genes before the 4-cell stage and in the divergent genes at the 8-cell stage onward (Supplementary information, Figure S14). The general pattern of NDRs in the ICM was very similar to that in mouse ES cells but differed from that in the TE (Figure 8E). Furthermore, we identified 133 657 differential NDRs between the ICM and TE (Figure 8E). These NDRs can be analysed by unsupervised hierarchical clustering of individual ICM and TE and separated from each other accurately at the single cell level (Figure 8F).

To provide additional insight into how DNA methylation and chromatin changes are linked during early development, we calculated the correlation coefficient among DNA methylation, chromatin accessibility and the corresponding gene's expression level at each developmental stage (Supplementary information, Figure S15A). We found that there is no correlation among these three “omic” parameters in mature oocytes. This is in agreement with the fact that transcription was globally silenced at this stage (Supplementary information, Figure S15A). After fertilization, a clear negative correlation between DNA methylation of promoter regions and the expression of the corresponding genes was established (Supplementary information, Figure S15A). Chromatin accessibility of the promoter region (200 bp upstream TSS and 100 bp downstream TSS) correlated positively with the corresponding gene's expression level from zygote to blastocyst stage throughout the whole of preimplantation development (Supplementary information, Figure S15A). Furthermore, we observed a strong negative correlation between DNA methylation and chromatin accessibility after fertilization, although this is gradually weakened due to the global demethylation of the genome (Supplementary information, Figure S15A). Next, we analyzed the relationship between that variance of promoter DNA methylation and of chromatin accessibility among individual cells at each developmental stage (Supplementary information, Figure S15B). In general, the majority of genes showed relatively homogeneous DNA methylation and homogeneous chromatin accessibility at their promoter regions among individual cells after the late zygote stage (Supplementary information, Figure S15B). As expected, oocytes were most homogeneous with the lowest variance of DNA methylation and chromatin accessibility among individual cells (Supplementary information, Figure S15B). Compared to oocytes, female pronuclei were much more heterogeneous for both DNA methylation and chromatin accessibility (Supplementary information, Figure S15B). This indicates that shortly after fertilization, female pronuclei undergo rapid but unsynchronized DNA demethylation and establishment of open chromatin. Moreover, at each preimplantation developmental stage analyzed, the genes with higher chromatin accessibility variance tended to have less DNA methylation variance, and vice versa (Supplementary information, Figure S15B). This indicated that the genes with heterogeneous promoter chromatin states among individual cells and the genes with heterogeneous promoter DNA methylation tend to fall into different gene sets during preimplantation development. To gain further insight into how DNA methylation and chromatin changes are linked, we analyzed the relationship between chromatin accessibility variance among individual cells and the corresponding DNA methylation levels (Supplementary information, Figure S16A). We found that the promoters with higher chromatin accessibility variance tended to have lower DNA methylation levels after fertilization (Supplementary information, Figure S16A). On the other hand, the promoters with higher DNA methylation variance tended to have lower chromatin accessibility (Supplementary information, Figure S16B).

### Nucleosome positioning, ploidy, DNA replication timing and copy number variation of mouse early embryos

We found that in addition to the strongly positioned pattern of nucleosomes in promoter regions, nucleosomes were preferentially located at the boundaries between introns and exons (Supplementary information, Figure S17A). This suggests that nucleosomes are specifically enriched at splicing sites and are periodically distributed during the chromatin remodeling after fertilization. We also analyzed the ploidy of the preimplantation embryos. The oocyte (with the first polar body removed) and the second polar body, which are known to be diploid and haploid, respectively, were used as controls (Supplementary information, Figure S17B). We found that the ploidy of both the male and female pronuclei were between haploid and diploid, suggesting that the initiation of DNA replication had already begun in these pronuclei at the PN3 to PN4 stage (Supplementary information, Figure S17B). Late zygotes and most cells from cleavage stage embryos were between diploid and tetraploid, suggesting that the majority were in either S or G2/M phase (Supplementary information, Figure S17B). We also analysed the timing of DNA replication in early embryos by analyzing the DNA replication domains identified from mouse ES cells. Interestingly, we found that in general, the leading replication regions of ES cells were also replicated earlier during S phase in individual blastomeres of mouse preimplantation embryos than the lagging regions (Supplementary information, Figure S17C, S17D, S17E and S17F). This indicates that a similar set of replication initiation sites are used in preimplantation embryos compared to ES cells.

To analyze CNV in early embryos, we used the same stringent criterion as in mouse ES cells. We found that in 23 out of 182 single blastomeres, one or more chromosomes displayed either gain or loss of copies (Supplementary information, Figure S18A). Moreover, we found 4 pairs (8 out of 23) of blastomeres showed gain and loss of copies at same chromosome regions within each pair (Supplementary information, Figure S18B), indicating the occurrence of aberrant mitotic aneuploidies during cleavage.

## Discussion

Recently, several single-cell epigenome sequencing technologies have been developed^10,11,12,13,14,15,16,17,18^. Single-cell multi-omics sequencing technologies including single cell Trio-seq have also been developed by our lab and others^19,20,21^. However, none of these approaches can concurrently analyze different layers of epigenomes from the same individual cell. Here we developed single-cell COOL-seq technology, which can simultaneously analyze chromatin accessibility, nucleosome positioning, DNA methylation, CNV and ploidy from the same individual cell with high sensitivity and coverage. To our knowledge, this is the first time that different layers of epigenetic regulations can be determined concurrently in the same single cell. We are able to detect both the degree of chromatin openness and endogenous DNA methylation levels for the promoter regions of majority (75.3%, 18 337 out of 24 346) of the RefSeq genes in a single ES cell. Our approach can also detect both the chromatin openness and endogenous DNA methylation levels for majority (70.3%, 11 246 out of 15 991) of the CpG islands from just a single ES cell. In contrast, the published single-cell epigenome sequencing technologies usually have only 1%-25% efficiency. Our method can simultaneously detect both the chromatin state and DNA methylation state of promoter regions for more than 70% of the RefSeq genes.

Considering the relatively low coverage of the single-cell epigenome sequencing techniques, it would be ideal if the closed chromatin state and the undetected state can be discriminated unambiguously. For example, if the detection sensitivity of such a technique is 20%, and a genomic locus has an open chromatin state in 50% of the cells in a population and a closed state in the remaining 50%, the method will call an open chromatin state in 10% of the cells, leaving the remaining 90% of the cells undetermined. Assuming that all of the remaining 90% of the cells have a closed chromatin state will lead to severe error of interpretation. However, if a method can discriminate between the closed chromatin state and the undetected state, it will call an open chromatin state in 10% of the cells and a closed chromatin state in another 10% of the cells, leaving the remaining 80% of the cells undetermined. In such a condition, the ratio of open and closed chromatin states can be accurately estimated as 1:1 (measured 10%:10% and corresponding to 50%:50% in real terms). To our knowledge, scCOOL-seq is the only single-cell chromatin state sequencing method that can achieve this. Moreover, scCOOL-seq can simultaneously detect the openness of the chromatin state, nucleosome positioning, endogenous DNA methylation, CNVs and ploidy in the same individual cell. This will greatly enhance the ability to analyze the complex relationships between these different genetic and epigenetic layers within an individual cell. Thus, the approach has potentially a wide range of applications to study physiological conditions such as embryonic development and pathological conditions such as tumorigenesis.

In summary, we have developed a single-cell multi-omics sequencing technology that can simultaneously determine chromatin status, nucleosome positioning, DNA methylation, CNV and ploidy from the same individual cell. This has allowed us, for the first time, to observe chromatin accessibility and DNA methylation on a genome-wide scale at single-cell and single-base resolution. At the same time, it has permitted such analyses in ES cells and in a parental allele-specific manner in mouse preimplantation embryos at seven critical developmental stages. We found that in each individual blastomere from the late zygote to 4-cell stage, the intergenic regions of paternal alleles are consistently hypermethylated compared with maternal alleles (Figure 8G). On the contrary, for intragenic regions, paternal alleles are hypomethylated compared with maternal alleles in each individual blastomere from late zygote stage and throughout preimplantation development (Figure 8G). However, the openness of chromatin of paternal and maternal alleles is essentially the same for both intragenic and intergenic regions in every individual blastomere (Figure 8G). This highlights the distinct patterns and functions of DNA methylation and chromatin states. Moreover, the binding motifs of several pluripotency master transcription factors showed stage-specific enrichment at distal NDRs (Figure 8G) and some were enriched from as early as the 2-cell stage. This indicates that the *cis*-regulatory elements of their target genes have been primed to an open state from the 2-cell stage onward, long before pluripotency is eventually established in the ICM at the blastocyst stage. We also classified genes into homogeneously open, homogeneously closed and divergent states based on the chromatin accessibility of their promoter regions among individual cells. We could trace their step-wise transitions from a homogeneously closed to a homogeneously open state via a divergent state through preimplantation development. We found that the homogeneously open genes were gradually established after fertilization and candidate genes that are important for developmental processes showed a divergent state at their promoters in the ICM. Our study offers an opportunity to understand the complex yet highly ordered epigenetic reprogramming of both DNA methylation and the chromatin state and their relationship with each other, as well as to gene expression when fully differentiated gametes are reprogrammed to a totipotent state and further to a pluripotent state to form later the embryo proper.

## Materials and Methods

### Animal use and care

Animal procedures were carried out according to the ethical guidelines of the Peking University Laboratory Animal Center.

### Cell culture and collection of mouse preimplantation embryos

The male mouse ES cell line (named F15, gift from Dr M Azim Surani) with the *Oct4-ΔPE-GFP* transgene was maintained without feeders on gelatinized dishes (3.5 cm) in 2i (3 μM CHIR99021 and 1 μM PD0325901) plus LIF (1 000 U/ml) media with 20% FBS (Gibco) for routine passage, as previously described^51^. For single-cell suspension, cells were digested with 0.05% trypsin and then washed in 1× DPBS plus 1 mg/ml BSA (Sigma, B8894). The “GMyc-PCR Mycoplasma Test Kit” (Yeasen, 40601ES10) was used to confirm absence of mycoplasma contamination.

To collect mouse preimplantation embryos, 6- to 8-week-old C57BL/6J females were superovulated by injection of 8-10 IU of PMSG, followed by injection of 8-10 IU of hCG 48 h later. MII oocytes were collected 14-15 h after hCG treatment. To collect embryos, females were mated with 129/sv males after hCG injection. Different stages of embryos were collected 28-29 h (late zygote), 43-44 h (late 2-cell), 56-57 h (4-cell), 70-71 h (8-cell), 78-79 h (morula) or 94-96 h (blastocysts) after hCG injection.

To inhibit transcriptional activity in mouse zygote, zygotes (about 4-5 h post fertilization) were incubated in KSOM medium containing 0.1 mM of α-Amanitin (Millipore, Cat# 129741-1MG) for another 5 h in a cell culture incubator (37 °C, 95% air and 5% CO_2_). RNA synthesis in mouse zygotes was analyzed by using the Click-iT RNA Imaging Kits (Invitrogen, Cat# C10329) following the supplier's instructions.

To isolate pronuclei from mouse zygotes, the zona was broken using a Piezo drive (Prime Tech) and male and female pronuclei (distinguished by their size and the distance from polar bodies) were harvested from zygotes (22-24 h after hCG injection) by aspiration using a micromanipulator. Female pronuclei were extracted afterward, as we previously reported^25,26,52^.

For the fluorescence immunostaining detection of protein in mouse preimplantation embryos, we followed the procedure as described in our previous publications^25,26,53^. The anti-Arnt monoclonal antibody (Cat# ab2771) was purchased from Abcam. Fluorescence images were acquired by using Leica TCS SP8 STED confocal microscope (Core Facilities of Life Sciences, Peking University).

### Preparation and *in vitro* methylation of single-cell nuclei

We first prepared a cell lysate, methylated the chromatin *in vitro*, released genomic DNA from the chromatin, and treated the genomic DNA with bisulfite in a one-tube reaction to avoid loss of material in the isolation and purification steps. Next, the bisulfite-treated genomic DNA from a single cell was amplified by a PBAT strategy to obtain sufficient material for sequencing. Briefly, a single cell or blastomere was picked using a mouth pipette and transferred into a 0.25 ml PCR tube containing 3.5 μl of ice-cold lysate buffer (50 mM Tris·HCl (pH 7.4), 50 mM NaCl, 10 mM dithiothreitol, 0.25 mM EDTA, 0.25 mM phenylmethylsulfonyl fluoride and 0.5% NP-40, plus 1 pg λDNA) and was kept on ice for 10 min. Then, the GpC methyltransferase M.CviPI and S-adenosylmethionine (New England Biolabs, M0227L) were added to the lysate to a final volume of 5 μl containing 1 U/μl M.CviPI and 160 μM SAM. *In vitro* methylation of single-cell nuclei was performed by incubating the mixture in a thermocycler at 37 °C for 30 min before heat inactivation for 20 min at 65 °C. After *in vitro* methylation, 0.5 μl of 20 mg/ml protease (Qiagen) was added and the mixture was incubated for 3 h at 50 °C to release genomic DNA. The released genomic DNA was then bisulfite converted and used to prepare the single-cell COOL-seq Library.

### *In vitro* methylation of nuclei from bulk cells and isolation of genomic DNA

In NOMe-seq, cells were mildly lysed to preserve chromatin structures, and then M.CviPI was used for *in vitro* methylation of both cytosine residues (C^5^) in a GpC dyad (5′GC3′/3′CG5′) in open chromatin accessible to the enzyme. Then, genomic DNA was purified and treated with bisulfite followed by whole-genome sequencing. NOMe-seq can simultaneously detect chromatin accessibility, nucleosome positioning and endogenous DNA methylation. The *in vitro* methylation of bulk cell nuclei was performed as previously described, but with some modifications^39,40,41,42,43^. Briefly, millions of sperm cells from mouse epididymides and trypsin-digested mouse ES cells (1–1.5 × 10^6^ cells) were first washed twice in 1 ml of 1× DPBS and centrifuged for 3 min at 500× *g* in a 4 °C microcentrifuge. The cells were then re-suspended in 500 μl of ice-cold 1× lysis buffer plus PMSF and protease inhibitors, and kept on ice for 20 min. Nuclei were prepared following the manufacturer's instructions of the nuclear preparation kit (Active Motif, 53504). To ensure cell lysis, 10 μl of the cell lysate was taken and visually checked under a phase contrast microscope. The nuclei were pelleted by centrifugation at 2 400× *g* for 10 min at 4 °C. After carefully removing the supernatant, nuclei were re-suspended with 1 ml of 1× M.CviPI reaction buffer (New England Biolabs). Approximately 1.0–1.5 × 10^5^ nuclei were methylated *in vitro* in the presence of 0.5 U/μl M.CviPI and 160 μM SAM for 45 min at 37 °C. After heat inactivation at 65 °C for 20 min, the nuclei were treated with 0.5 mg/ml proteinase K in an equal volume of 2× digestion buffer (20 mM Tris·HCl (pH 7.4), 200 mM NaCl, 2% SDS and 10 mM EDTA) at 55 °C overnight. To isolate sperm DNA, 40 mM DTT was added to the digestion mixture. The DNA was extracted with phenol-chloroform, followed by ethanol precipitation. Then, 50-100 ng of genomic DNA was used to construct the PBAT library for whole-genome bisulfite sequencing.

### Single-cell COOL-seq library preparation and sequencing

After *in vitro* methylation of the single-cell nuclei with M.CviPI, the genomic DNA released by proteinase treatment was used to construct the COOL-seq library using a single-cell PBAT strategy, as previously described^16,53^. Briefly, the single-cell genomic DNA was bisulfite converted using the MethylCode Bisulfite Conversion Kit (Invitrogen) following the manufacturer's instructions. Then, the purified DNAs were annealed using random nonamer primers with a 5′-biotin tag (5′-Biotin-CTACACGACGCTCTTCCGATCTNNNNNNNNN-3′) in the presence of Klenow fragments (3′-5′ exo-, New England Biolabs). Then, the primers were digested by exonuclease I (NEB) and the DNA was purified using Agencourt Ampure XP beads (Beckman Coulter). Dynabeads M280 (Invitrogen, streptavidin-coupled) were then used to immobilize the newly synthesized biotin-tagged DNA strands, and the original bisulfite-converted DNA templates were removed. Second DNA strands were synthesized using Klenow fragments with random nonamer primers (5′-AGACGTGTGCTCTTCCGATCTNNNNNNNNN-3′). After washing, the beads were used to amplify libraries using 13 cycles of PCR with the Illumina Forward PE1.0 primer and Illumina Reverse indexed primer (New England Biolabs) in the presence of Kapa HiFi HS DNA Polymerase (Kapa Biosystems). The amplified libraries were purified with Agencourt Ampure XP beads twice and were assessed on the Fragment Analyzer (Advanced Analytical Technologies). Finally, libraries were pooled (quantified with qPCR) and sequenced on the Illumina HiSeq 2500 sequencer for 150 bp paired-end sequencing.

### Validation of open and closed chromatin in scCOOL-seq by liDNaseI-qPCR assay

About 100 ES cells were treated by following the published liDNaseI-qPCR protocol^34^. Four biological replicates were performed for each locus and the sequences of qPCR primer pairs were as listed below (5′-3′):

*Oct4*: sense 5′-CCTAAGGGTTGTCCTGTCCAGA-3′,

antisense 5′-CTAGGGACGGTTTCACCTCTCC-3′

*Nanog*: sense 5′-GTCACCTTACAGCTTCTTTTGCAT-3′,

antisense 5′-GCTCAAGGCGATAGATTTAAAGGG-3′

*Uhrf1*: sense 5′-GTGGGGTAGATCCTTAGTCATGC-3′,

antisense 5′-ACTCAGGGCGTTTTTATTAGTGTG-3′

*Tbx3*: sense 5′-TGATCATGTTGACATAAACGCAGG-3′,

antisense 5′-TTGATTGGCTCTTTGACGCTTTC-3′

*Klf5*: sense 5′-TGATTTCCCCCTCTTCCTAGATTC-3′,

antisense 5′-AGAGGGTAGCCAGTAGGAAAGAA-3′

*Zfp53*: sense 5′-GCTGCGTCATATCAGATCCAGTTC-3′,

antisense 5′-AATGTGTAACATCCTCCCATCCTC-3′

Closed Locus 1: sense 5′-GTCAACCTTCTACAGTGATCCTCC-3′,

antisense 5′-GTAAGTTCTGCAGTCCTCCTGTA-3′

Closed Locus 2: sense 5′-CAGCAGCAGCTGATATGGACA-3′,

antisense 5′-GTGAATTCCTTTGCTCCGAGGT-3′

### Single-cell COOL-seq data processing

**Quality control and read mapping of single-cell COOL-seq data** Raw reads were trimmed to remove the first 9 bases and to remove the adapter-contaminated and low-quality reads using Trim Galore (v0.3.3). The cleaned and QC-ensured reads were then aligned to the *in-silico* bisulfite-converted mouse genome reference (mm9) using Bismark (v0.7.6) with paired-end and non-directional mapping parameters^54^. After paired-end mapping, the unmapped reads were re-aligned to the same reference genome in single-end mode. Duplicated reads from the PCR amplification step were identified and removed by using their genomic coordinates under published SAMtools (v0.1.18) following the “samtools rmdup” command (v0.1.18) parameter: “samtools rmdup” for paired-end reads and “samtools rmdup -s” for single-end reads. Only the non-duplicated reads were further used for the downstream analysis including CNV analysis^55^.

**Quantification of WCG and GCH methylation level** The DNA methylation level of each covered cytosine was calculated as the “methylated” reads (reported as C) divided by the total number of “methylated” and “unmethylated” reads (reported as C or T) at the same reference position. For all downstream analyses, both GCG and CCG trinucleotides were excluded, as the on-target M.CviPI activity on GCGs and the slight off-target activity on CCGs previously described^39,43^ affect *in vivo* CG methylation. For each individual cell, every covered WCG site (W includes A or T) and GCH site (H includes A, C or T) with at least one-fold coverage (≥ 1× depth) was summed. The average DNA methylation level and chromatin accessibility of the samples were estimated as the average WCG and GCH level, respectively. For bulk cell samples, every covered WCG site or GCH site with at least three-fold coverage was summed. For the majority of analysis of DNA methylation or chromatin accessibility among individual cells, WCG or GCH sites with at least 2× depth were used.

We determined how many CpG and GpC sites could be recovered from our single-cell COOL-seq data. We analyzed 24 individual mouse ES cells and identified the presence of 2 217 720-4 955 081 unique CpG sites in each individual cell. Our approach recovered on average 3.8 million (10.6%) of the total 36 167 049 CpG sites that could be detected in bulk ES cells. At the same time, we detected on average 16.3 million (9.4%) (9 508 821-21 656 501) of the 174 770 340 unique GpC sites in each individual cell. When we merged the scCOOL-seq data for 24 individual cells together *in silico*, many more CpG and GpC sites were recovered. There were in total 28 831 281 (79.7%) unique CpG and 134 973 288 (77.2%) unique GpC sites recovered in the merged single-cell data set.

For each individual ES cell, we could recover on average 12.5% (range from 7.5% to 16.5%) of the genome when each single-cell sample was sequenced at ∼2× (6.0 Gb) coverage. Furthermore, we could detect on average 11 246 (70.3%) of 15 991 CpG islands (covered at least five GCH sites in scCOOL-seq) and cover the promoter regions of 18 337 (75.3%) of 24 346 RefSeq genes for each individual ES cell. When the data from all of the 24 single ES cells were merged, we could cover 77.6% of the genome and cover the promoter regions of 24 095 (99.0%) of the 24 346 RefSeq genes when the samples were sequenced at 56× (∼150 Gb data in total; 5.2× depth for each sample on average). Moreover, we could detect 28.8 million (67.4%) unique CpG sites.

**Genomic region annotation** CpG island (CGI) information was downloaded from the UCSC Genome Browser (mm9), and all the repetitive elements information, such as LINE, SINE and LTR elements and their subfamilies, were downloaded from the mm9 Repeat Masker. Promoters were defined as the regions of 1 kb upstream and 0.5 kb downstream of the TSS and were classified into HCP (high-density CpG promoter), ICP (intermediate-density CpG promoter) and LCP (low-density CpG promoter) as previously described^14,52,53^.

**Analysis of de novo methylated WCG sites and CGIs in mouse early embryos** To identify the *de novo* methylated WCG sites in early embryos, we first extracted the WCG sites, which were covered in at least three single cells within each stage and calculated the mean methylation level among the covered single cells in the same stage. Then, we used the following cutoff to identify *de novo* methylated sites: a WCG site with a < 0.25 methylation level at one stage and with at least a 0.3 methylation level increase in the next stage (Benjamini-Hochberg's FDR < 0.05) was defined as a *de novo* methylated WCG site. As a control, we also calculated the demethylated WCG sites in the early embryos using the cutoff: a WCG site with a methylation level over 0.75 in the former stage and with at least a 0.3 methylation level decrease in the next stage was defined as a demethylated WCG site. Because the most significant enrichment in *de novo* methylated WCG sites was in CGIs, we calculated how many CGIs were *de novo* methylated after fertilization using the following cutoff: a CGI (which was detected at least three WCG sites in a single cell and covered in at least three single cells within each stages) with a < 0.25 average methylation level in one stage and with at least a 0.3 increase in methylation level in the next stage was defined as a *de novo* methylated CGI. We found that *de novo* methylation dominated from the late zygote stage to the 4-cell stage because the number of *de novo* methylated WCG sites was much higher than that of demethylated sites during this developmental period, resulting in an increase in the global methylation level from the late zygote stage to the 4-cell stage (zygote versus 4-cell, *P* = 1.6 × 10^−3^).

**Defining NDRs and nucleosome occupied regions** First, merged single-cell COOL-seq data within each stage were used. The number of C and T nucleotides sequenced was counted at each GCH site in the genome. To define the NDR, the C and T counts were summed in 120-bp windows at 20-bp spacing and tested for differences from the genomic background using the *χ*^2^-test. Significant highly GCH methylated windows were retained if the *P*-value ≤ 10^−15^, then overlapped and only retained as NDRs if they were a minimum of 140 bp in size and covered at least five GCH sites according to the previous publication of NOMe-seq^39,40,43^. Each NDR was further separated into two categories: distal (at least 2 kb away from the TSS) and proximal (within 2 kb upstream and downstream of the TSS) NDRs, respectively.

For defining the nucleosome occupied regions, the C and T counts were summed in 40-bp windows at 20-bp spacing and tested for differences from the genomic background using the *χ*^2^-test. Significant minimally GCH methylated windows were retained if *P*-value ≤ 10^−3^, then overlapped and only retained as nucleosome-occupied regions if they were a minimum of 60 bp in size, with at least three GCH sites covered.

**Combinatorial analysis of epigenomic signatures in mouse ES Cells** To perform comparison analysis of our single-cell COOL-seq data from mouse ES cells with published data sets describing histone modification, transcription factor occupation, DNase I hypersensitive sites and nucleosome positioning, we used the following published ChIP-seq, DNaseI-seq and MNase-seq data sets from mouse ES cells from Gene Expression Omnibus (GEO): GSE29184 for H3K4me3, H3K4me1, H3K27ac, CTCF, EP300 and RNA Pol II ChIP-seq; GSE11431 for Oct4 ChIP-seq; GSE62380 for H3K27me3 and H3K9me3 ChIP-seq; GSE37074 for DNaseI-seq and GSE69098 for MNase-seq.

**Motif analysis of NDRs** For motif analysis of NDRs detected in either bulk cells or single cells, a published tool named findMotifsGenome.pl in HOMER v4.7.2 was used to analyze motif enrichment in the distal and proximal NDRs of each cluster. The parameters “-size 2000 -len 8 -S 100” were applied. Motifs with the *P*-value below 10^−12^ were considered as significantly enriched.

**Principal component analysis of DNA methylation across individual blastomeres** To assess the cell populations of all the single-cell COOL-seq samples, principal component analysis was performed using the DNA methylation level (WCG level) of all 5-kb tiles, which were covered at least five WCG sites. And the pcaMethods package in R was used to analyze the data.

**Single-cell CNV analysis** For CNV deduction with single-cell COOL-seq data, we used the published R package HMMcopy^56^. Briefly, we used readCounter to bin the genome into consecutive 1 Mb windows and calculated the absolute number of reads detected in each window. Then, we used HMMcopy to estimate the copy number with GC and mappability corrections. The median values of each window from 24 single ES cells were used to normalize the samples. The CV was calculated in each sample to evaluate the deviation of our single-cell COOL-seq in analyzing the CNV. We found that even at as small as 1 Mb resolution, the measurements were highly accurate and robust, with the CV as low as 0.11 (between 0.07 and 0.16) among individual mouse ES cells. The CNV analysis of preimplantation embryos was under the same criteria as that in mouse ES cells.

**Single-cell ploidy analysis** To infer the single-cell ploidy from the single-cell COOL-seq data, we spiked the same quantity of λDNA into each single-cell sample to deduce the ploidy of the cell. For the mouse single oocytes, second polar bodies or blastomeres, we spiked ∼1 pg λDNA to each single-cell sample. The ratio of mouse genomic DNA reads to lambda DNA reads was calculated after processing the raw data from scCOOL-seq. We found that for the 24 single ES cells we analyzed, three (12.5%) were estimated diploid (2N), which likely indicates that they were in the G1 phase. Five (20.8%) were estimated tetraploid (4N), which may indicate that they were in the G2/M phase, and 16 (66.7%) were estimated between 2N and 4N, which likely indicates that they were in S phase. These data are compatible with the fact that 20.3%-25.3%, 51.2%-62.0% and 16.8%-28.5% of ES cells are at G1, S and G2/M phase, respectively, as reported by different groups. Next, we calculated the ratio of genomic reads to λ DNA reads in each single oocyte (known to be diploid after the first polar body has been extruded), and defined the mean value of all the ratios calculated in MII oocytes, which were assumed to be diploid (2N). Then, the ratio calculated in each single cell was normalized by the assumed diploid value calculated from the oocytes. The second polar body, which is known to be haploid (1N), was also calculated as haploid based on comparison to the data from the oocytes in this study, suggesting the accuracy of our ploidy analysis. Both the male and female pronuclei were estimated to be between haploid (1N) and diploid (2N), which was consistent with the fact that the pronuclei initiate DNA replication near the PN3 stage after fertilization.

**DNA replication timing of mouse ES cells** To confirm our CNV and ploidy analysis result in single mouse ES cells, we first downloaded the published data describing mouse ES DNA replication timing, which contain the mean replication-timing ratios log_2_(Early/Late) for all microarray probes (GSE49847)^57,58,59^, then used DNAcopy (R package) to identify replication domains with parameters (*alpha* = 10^−15^, *nperm* = 10 000) that are described in the original study^59^. There were in total 25 150 replication domains identified in the mouse ES cells, ranging from 5 kb to 7 Mb in size (95 kb on average). The replication domains with mean replication-timing ratios above 1.0 were defined as leading regions during S phase (*n* = 6 394), whereas the replication domains with mean replication-timing ratios below 0 were defined as lagging regions during S phase (*n* = 12 877). Next, we analyzed the single-cell COOL-seq data from 24 individual ES cells and used readCounter to bin the genome into consecutive 100 kb tiles, then calculated the absolute number of reads detected in each window. After correction and normalization using HMMcopy, we calculated the averaged read counts of 100 kb tiles that overlapped with DNA replication domains in each single ES cell. The mean values of counts in the leading and lagging replication regions were calculated for each single-cell sample. A two-tailed t-test was used to calculate the statistical significance of copy number differences between the leading and lagging regions.

It has been shown that the replication timing of different genomic regions in ES cells is different, with some heterochromatin regions lagging during DNA replication in S phase^58,59,60^. We found that for the 16 individual cells we proposed to be in S phase, the leading genomic regions tended to have more copies than the lagging ones. As a control, for the G2/M-phase ES cells, the copy numbers of the leading genomic regions were comparable to those of the lagging ones, as expected. This suggests that both CNV measurement and ploidy measurement of our method are accurate, and that we could use them to deduce the cell cycle stage of the single cells we analyzed. To our knowledge, this is the first time that the ploidy of a single cell has been measured using a single-cell sequencing strategy, as for all of the previous single-cell genome sequencing techniques, a cell was assumed to be either diploid or haploid.

**Allele-specific analysis of parental genomes in mouse preimplantation embryos** To track the DNA methylation and chromatin accessibility of both paternal and maternal genomes by heterozygous SNPs between 129sv (paternal) and C57BL/6J (maternal) mice at single-cell resolution, we downloaded the 5 853 614 SNPs between 129sv and C57BL/6J from the public database of the Sanger Institute (http://www.sanger.ac.uk/science/data/mouse-genomes-project). First, to validate our analysis pipeline used for SNP tracing, we processed sequenced reads from scCOOL-seq of mouse sperm, which were derived from the 129sv background. By using this data from mouse sperm, we obtained 2 053 100 and 51 791 WCG sites traced by 129sv and C57BL/6J SNPs, respectively (the ratio is 40:1). Meanwhile, we obtained 17 856 223 and 444 774 GCH sites traced by 129sv and C57BL/6J SNPs, respectively (the ratio is 40:1). On the contrary, when we used the scCOOL-seq data from mouse oocyte, which were derived from C57BL/6J background, we obtained 1 286 921 and 18 297 WCG sites traced by C57BL/6J SNPs and 129sv, respectively (the ratio is 70:1). 11 200 018 and 151 931 GCH sites traced by C57BL/6J SNPs and 129sv SNPs were obtained, respectively (the ratio is 74:1). These results indicated high accuracy of our pipeline for SNP tracing. Next, we used this established pipeline to track parental genomes across preimplantation development. On average, we obtained hundred thousands of WCG sites (≥ 1× depth) from both parental genomes in each individual cell, and millions of GCH sites (≥ 1× depth) from both parental genomes in each individual cell. These sites from both parental genomes within individual cells were used for downstream analysis.

**Analysis of the heterogeneity of promoter accessibility among individual cells** To classify the promoters according to the chromatin accessibility among individual cells, we performed the analysis as follows: First, we identified promoter NDRs (NDRs contain TSS) in the merged single cells (for example 9 685 promoter NDRs were defined in the merged single ES cells). Next, we used the promoter NDRs identified in the merged single ES cell data to measure the chromatin status (open or closed) among individual cells. Because the averaged GCH methylation level of an NDR was above 0.5, while the averaged GCH methylation level of a nucleosome was always below 0.3. We used the cutoff for defining chromatin status in a single cell as this: if a promoter NDR defined in merged single cell samples is covered in a single cell (at least five GCH sites covered) with the averaged GCH methylation level above 0.5, this region is defined as open chromatins in this single cell; If a promoter NDR defined in merged single cell samples is covered in a single cell (at least five GCH sites covered) with the averaged GCH methylation level below 0.3, this region is defined as closed chromatin in this single cell. For those promoters with no NDRs detected in the merged single cells, the regions of 200 bp upstream TSS and 100 bp downstream TSS were used for analysis. And the chromatin status in these promoter regions among individual cells were defined as above. Then, we extracted the promoters that covered in at least half numbers of the total sequenced cells within each stage (for example those promoters covered by at least 12 cells in sequenced single ES cells were extracted), and calculated the number of cells defined as open or closed status in these promoters. If over 70% number of cells were defined as having open status in a promoter, this promoter was defined as a homogeneously open promoter; if over 70% number of cells were defined as having closed status in a promoter, this promoter was defined as a homogeneously closed promoter; if between 30% and 70% number of cells were defined as having open status in a promoter, this promoter was defined as a divergent promoter.

To validate our classification, we performed additional single-cell RNA-seq of mouse ES cells (16 ES cells by using the Smart-seq2 method) and downloaded the mouse single-cell RNA-seq data from preimplantation embryos (zygote, late 2-cell, 4-cell, 8-cell, 16-cell and blastocyst) published by Rickard Sandberg's lab^61^ (accession number: GSE45719). We found that the homogeneously closed genes of ES cells were not expressed, and the homogeneously open genes were actively transcribed with lowest coefficient of variance in their expression compared to the divergent and closed genes, as expected.

For the GO analysis of genes within each stage, GOstats package in the Bioconductor R program was used and all genes that defined homogeneously open, homogeneously closed and divergent states within this stage were set as the background.

### Data accession

A total of ∼2.0 Tb sequencing data (including 282 of single-cell COOL-seq libraries, 13 of bulk cells NOMe-seq libraries, 15 of titration series COOL-seq libraries, 16 of single-cell RNA-seq libraries and 3 of bulk cells RNA-seq libraries) were generated for this work. All the sequencing data were deposited in the NCBI GEO under accession number GSE78140.

## Author Contributions

FG and FT conceived the project. FG, JL and XW performed the experiments. LL, BH and PZ conducted the bioinformatics analyses. FG, LW and FT wrote the manuscript with help from all of the authors.

## Competing Financial Interests

The authors declare no competing financial interests.

## Supplementary Information

Supplementary information, Figure S1 Chromatin accessibility of individual mouse ES cells around the transcription start site (TSS) revealed by single-cell COOL-seq analysis.

Supplementary information, Figure S2 Accuracy and sensitivity of the single-cell COOL-seq technique.

Supplementary information, Figure S3 The features of chromatin accessibility detected in bulk ES cells can be robustly reproduced in merged single ES cells.

Supplementary information, Figure S4 Single-cell COOL-seq analysis reveals epigenomic signatures of chromatin accessibility in mouse ES cells.

Supplementary information, Figure S5 Nucleosome positioning patterns observed by single-cell COOL-seq method.

Supplementary information, Figure S6 Representative loci detected as either open or closed chromatin by single-cell COOL-seq method were validated by liDNaseI-qPCR assay.

Supplementary information, Figure S7 Robust and accurate detection of NDRs and nucleosomes across individual ES cells.

Supplementary information, Figure S8 Variation of DNA methylation and chromatin accessibility at specific genomic elements among different individual cells at each developmental stage.

Supplementary information, Figure S9 Chromatin accessibility of mouse preimplantation embryos revealed by single-cell COOL-seq analysis.

Supplementary information, Figure S10 Chromatin accessibility and DNA methylation at promoters, NDRs and nucleosomes during preimplantation development.

Supplementary information, Figure S11 Dynamics of chromatin accessibility of different functional genomic elements in mouse early embryos.

Supplementary information, Figure S12 Dynamics of chromatin accessibility of subfamilies of SINEs.

Supplementary information, Figure S13 Dynamic of DNA methylation and chromatin accessibility of parental genomes within individual cells in preimplantation embryos.

Supplementary information, Figure S14 Heterogeneity analysis of promoter accessibility in preimplantation embryos.

Supplementary information, Figure S15 The relationship among DNA methylation, chromatin accessibility and expression of RefSeq genes during mouse preimplantation development.

Supplementary information, Figure S16 The relationship between DNA methylation and chromatin accessibility during mouse preimplantation development.

Supplementary information, Figure S17 Nucleosome positioning, ploidy and DNA replication timing of mouse early embryos.

Supplementary information, Figure S18 Copy number variations in mouse preimplantation embryos.

Supplementary information, Table S1 Summary of single-cell Cool-seq data.

Supplementary information, Table S2 Motif enrichment analysis.

Supplementary information, Table S3 Classification of Gene Promoters.

Supplementary information, Data S1 Single-cell COOL-seq Protocol
