# Supplementary material for: Single-cell multi-omics sequencing of mouse early embryos and embryonic stem cells
Source: Cell Res. 2017 Jun 16;27(8):967–88. doi: 10.1038/cr.2017.82 (PMC5539349; doi:10.1038/cr.2017.82)
Supplement: Supplementary information, Figure S1 — Chromatin accessibility of individual mouse ES cells around the transcription start site (TSS) revealed by single-cell COOL-seq analysis. [file cr201782x1.pdf]

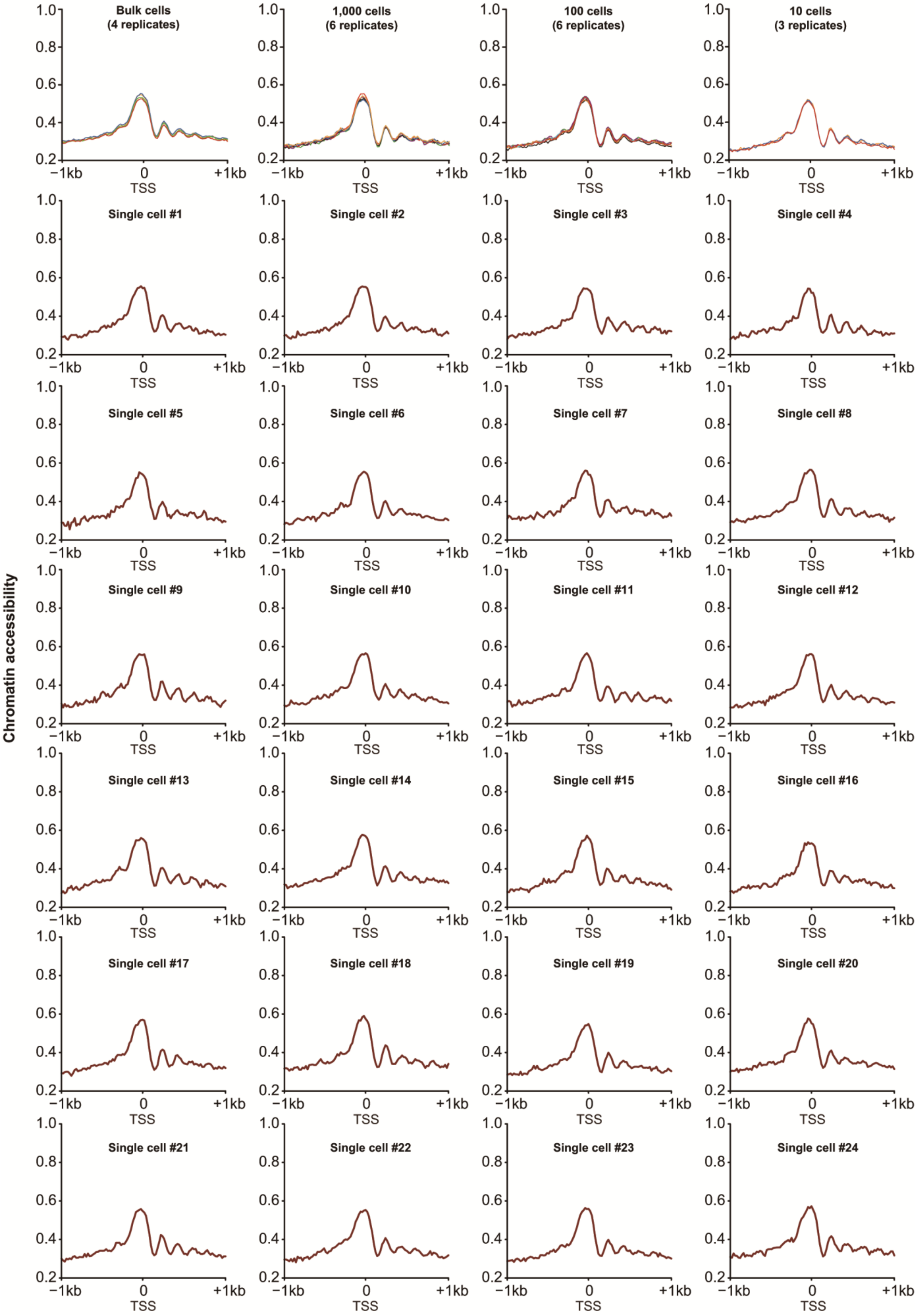
Chromatin accessibility
Bulk cells
(4 replicates)
1,000 cells
(6 replicates)
100 cells
(6 replicates)
10 cells
(3 replicates)
Single cell #1
Single cell #2
Single cell #3
Single cell #4
Single cell #5
Single cell #6
Single cell #7
Single cell #8
Single cell #9
Single cell #10
Single cell #11
Single cell #12
Single cell #13
Single cell #14
Single cell #15
Single cell #16
Single cell #17
Single cell #18
Single cell #19
Single cell #20
Single cell #21
Single cell #22
Single cell #23
Single cell #24
1.0
0.8
0.6
0.4
0.2
−1kb
0
+1kb
TSS

**Supplementary information, Figure S1.** Chromatin accessibility of individual mouse ES cells around the transcription start site (TSS) revealed by single-cell COOL-seq analysis. The chromatin accessibility (average GCH methylation levels) of bulk cells (4 replicates), titration series (from 1,000 cells to 10 cells) or 24 individual ES cell.
