# Supplementary material for: Single-cell multi-omics sequencing of mouse early embryos and embryonic stem cells
Source: Cell Res. 2017 Jun 16;27(8):967–88. doi: 10.1038/cr.2017.82 (PMC5539349; doi:10.1038/cr.2017.82)
Supplement: Supplementary information, Figure S2 — Accuracy and sensitivity of the single-cell COOL-seq technique. [file cr201782x2.pdf]

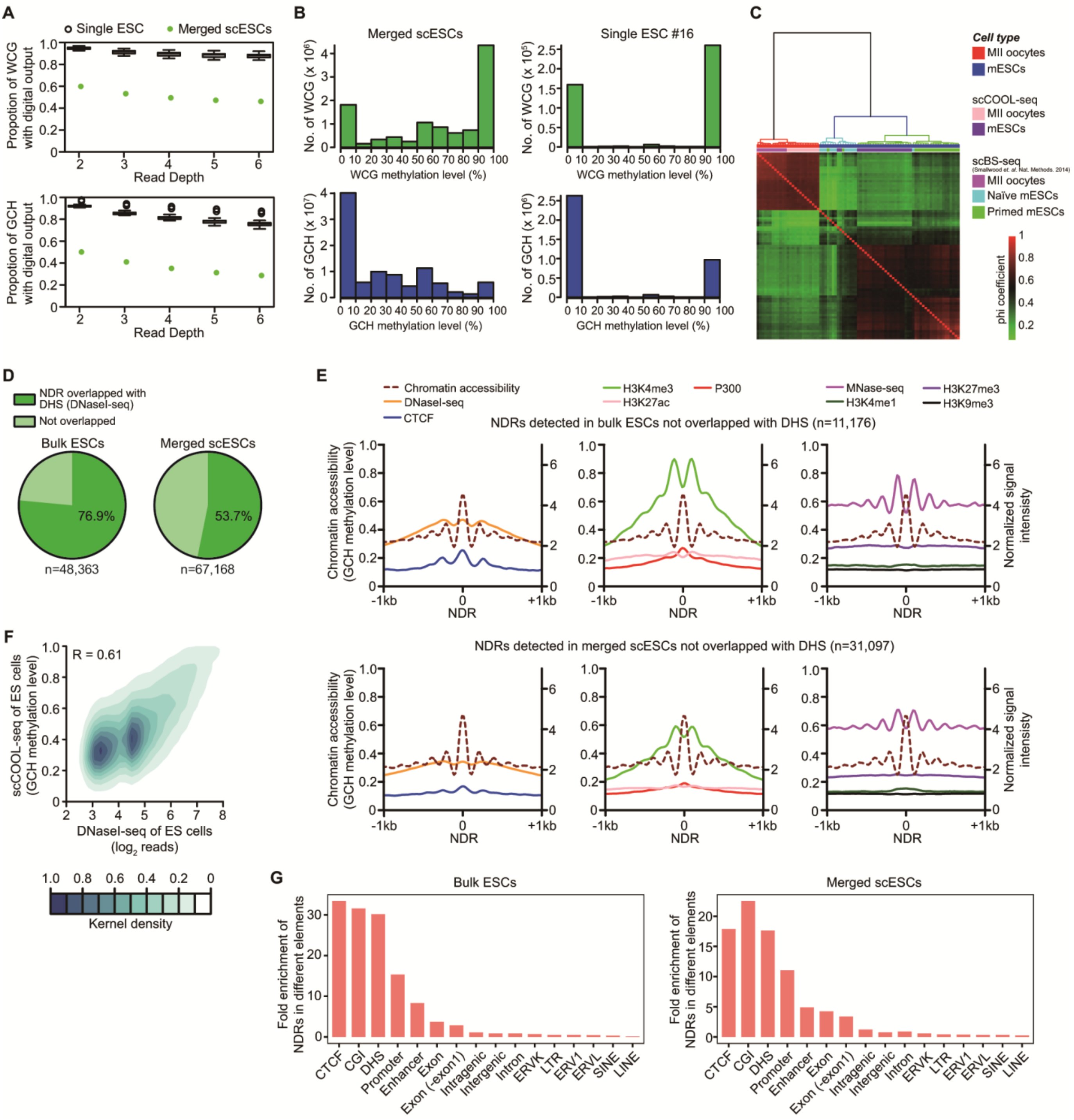

A
Single ESC
Merged scESCs
Propotion of WCG with digital output
Read Depth
Propotion of GCH with digital output
B
Merged scESCs
Single ESC #16
WCG methylation level (%)
GCH methylation level (%)
C
Cell type
MII oocytes
mESCs
scCOOL-seq
MII oocytes
mESCs
scBS-seq
MII oocytes
Naïve mESCs
Primed mESCs
phi coefficient
D
NDR overlapped with DHS (DNaseI-seq)
Not overlapped
Bulk ESCs
Merged scESCs
76.9%
53.7%
n=48,363
n=67,168
E
Chromatin accessibility
DNaseI-seq
CTCF
H3K4me3
H3K27ac
P300
MNase-seq
H3K4me1
H3K27me3
H3K9me3
NDRs detected in bulk ESCs not overlapped with DHS (n=11,176)
NDRs detected in merged scESCs not overlapped with DHS (n=31,097)
Chromatin accessibility (GCH methylation level)
Normalized signal intensisty
NDR
F
R = 0.61
scCOOL-seq of ES cells (GCH methylation level)
DNaseI-seq of ES cells (log2 reads)
Kernel density
G
Bulk ESCs
Merged scESCs
Fold enrichment of NDRs in different elements
CTCF
CGI
DHS
Promoter
Enhancer
Exon
Exon (-exon1)
Intragenic
Intergenic
Intron
ERVK
LTR
ERV1
ERVL
SINE
LINE

Supplementary information, Figure S2. Accuracy and sensitivity of the single-cell COOL-seq technique.

(A) Proportion of digitally detected WCG or GCH sites (either fully methylated or fully unmethylated) in each of the 24 individual ES cells.

(B) Histograms of the distribution of WCG or GCH methylation values for merged scESCs and single ES cell ≥ 2× depth.

(C) Phi coefficient of the DNA methylation level of single ES cells and single MII oocytes generated by scCOOL-seq with a published dataset of DNA methylation of single mouse ES cells and MII oocytes generated by scBS (Smallwood *et al.*, *Nat Methods*, 2014). In total, 13,074,422 single WCG sites (WCG sites that were detected at ≥ 2× depth in at least one single cell) were used to perform this analysis.

(D) The proportion of NDRs detected by single-cell COOL-seq analysis that overlapped with the DNA hypersensitive sites (DHS) in bulk ES cells detected by DNaseI-seq (175,237 of DHS sites in total, 150 bp length on average).

(E) Features of NDRs that were detected in bulk and single ES cells but did not overlap with the DHS sites from DNaseI-seq. The upper panel shows that the NDRs detected in bulk ES cells that did not overlap with DHS sites were enriched for H3K4me1 and H3K4me3 marks and depleted of nucleosomes. The lower panel shows that the NDRs that were detected in single ES cells but did not overlap with DHS sites were also clearly enriched for H3K4me1 and H3K4me3 marks and depleted of nucleosomes.

(F) Pearson correlation of global chromatin accessibility profiles between scCOOL-seq and published DNaseI-seq data. A total number of 97,541 DNaseI hypersensitive sites (DHS, narrow peaks) were used, and these DHS were detected in our merged scCOOL-seq containing at least 5 GCH sites with at least 5× sequencing depth.

(G) Distribution of the NDRs detected in bulk ES cells and single ES cells among different functional genomic elements. The DHS sites, CTCF binding regions and enhancers that were previously identified in bulk mouse ES cells were used (Hnisz *et al.*, *Cell*, 2013; Yue *et al.*, *Nature*, 2014).

References

Hnisz D, Abraham BJ, Lee TI, *et al.* Super-enhancers in the control of cell identity and disease. *Cell* 2013; **155**:934-947.

Smallwood SA, Lee HJ, Angermueller C *et al.* Single-cell genome-wide bisulfite sequencing for assessing epigenetic heterogeneity. *Nat methods* 2014; **11**:817-820.

Yue F, Cheng Y, Breschi A, *et al.* A comparative encyclopedia of DNA elements in the mouse genome. *Nature* 2014; **515**:355-364.
