# Supplementary material for: Single-cell multi-omics sequencing of mouse early embryos and embryonic stem cells
Source: Cell Res. 2017 Jun 16;27(8):967–88. doi: 10.1038/cr.2017.82 (PMC5539349; doi:10.1038/cr.2017.82)
Supplement: Supplementary information, Figure S3 — The features of chromatin accessibility detected in bulk ES cells can be robustly reproduced in merged single ES cells. [file cr201782x3.pdf]

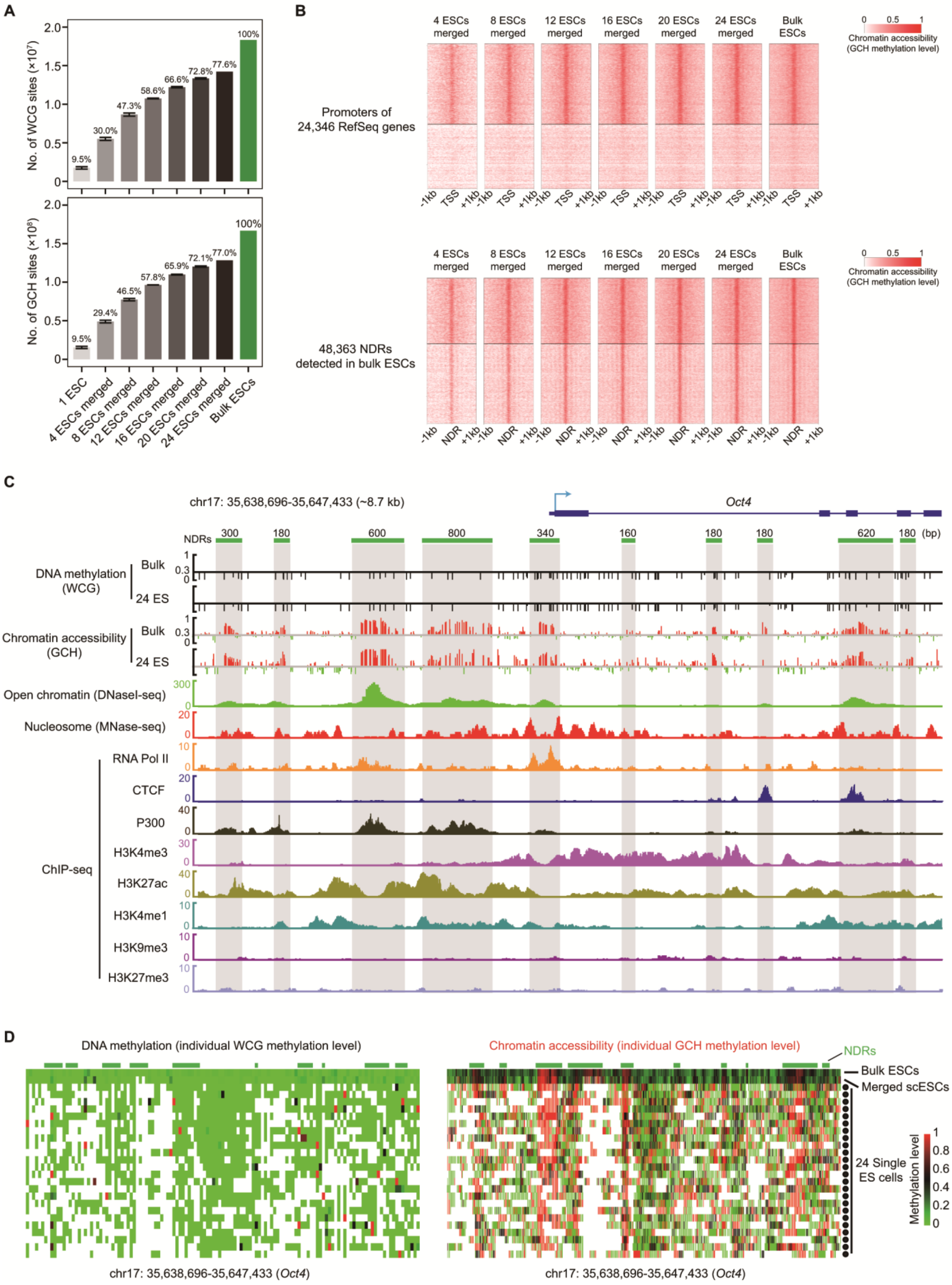

A
No. of WCG sites (×10⁷)
2.0
1.5
1.0
0.5
0
9.5%
30.0%
47.3%
58.6%
66.6%
72.8%
77.6%
100%
No. of GCH sites (×10⁸)
9.5%
29.4%
46.5%
57.8%
65.9%
72.1%
77.0%
100%
1 ESC
4 ESCs merged
8 ESCs merged
12 ESCs merged
16 ESCs merged
20 ESCs merged
24 ESCs merged
Bulk ESCs
B
4 ESCs merged
8 ESCs merged
12 ESCs merged
16 ESCs merged
20 ESCs merged
24 ESCs merged
Bulk ESCs
Chromatin accessibility (GCH methylation level)
0
0.5
1
Promoters of 24,346 RefSeq genes
-1kb
TSS
+1kb
48,363 NDRs detected in bulk ESCs
-1kb
NDR
+1kb
C
chr17: 35,638,696-35,647,433 (~8.7 kb)
Oct4
NDRs
300
180
600
800
340
160
180
180
620
180
(bp)
DNA methylation (WCG)
Bulk
24 ES
Chromatin accessibility (GCH)
Bulk
24 ES
Open chromatin (DNaseI-seq)
Nucleosome (MNase-seq)
ChIP-seq
RNA Pol II
CTCF
P300
H3K4me3
H3K27ac
H3K4me1
H3K9me3
H3K27me3
D
DNA methylation (individual WCG methylation level)
Chromatin accessibility (individual GCH methylation level)
NDRs
Bulk ESCs
Merged scESCs
24 Single ES cells
Methylation level
1
0.8
0.6
0.4
0.2
0
chr17: 35,638,696-35,647,433 (Oct4)
chr17: 35,638,696-35,647,433 (Oct4)

Supplementary information, Figure S3. The features of chromatin accessibility detected in bulk ES cells can be robustly reproduced in merged single ES cells.

(A) Bar plot of total WCG sites or GCH sites detected in single ES cells and merged single ES cells.

(B) Heat map shows that both the chromatin accessibility of promoters and NDRs observed in bulk ES cells can be robustly reproduced in merged single ES cells.

(C) DNA methylation level (WCG methylation level) and chromatin accessibility (GCH methylation level) at the *Pou5f1* (also known as *Oct4*) gene locus. The DNA methylation and chromatin accessibility were measured by single-cell COOL-seq. Open chromatin from published DNaseI-seq, nucleosome positionings from published MNase-seq and histone marks from ChIP-seq datasets were used as controls. NDR regions are highlighted with green bars.

(D) The DNA methylation level and chromatin accessibility in each of the 24 individual ES cells at single-base resolution. The color scale from green to red indicates the methylation level from low to high. The WCG or GCH site which was undetected in a single cell was marked with blank.
