# Supplementary material for: Single-cell multi-omics sequencing of mouse early embryos and embryonic stem cells
Source: Cell Res. 2017 Jun 16;27(8):967–88. doi: 10.1038/cr.2017.82 (PMC5539349; doi:10.1038/cr.2017.82)
Supplement: Supplementary information, Figure S4 — Single-cell COOL-seq analysis reveals epigenomic signatures of chromatin accessibility in mouse ES cells. [file cr201782x4.pdf]

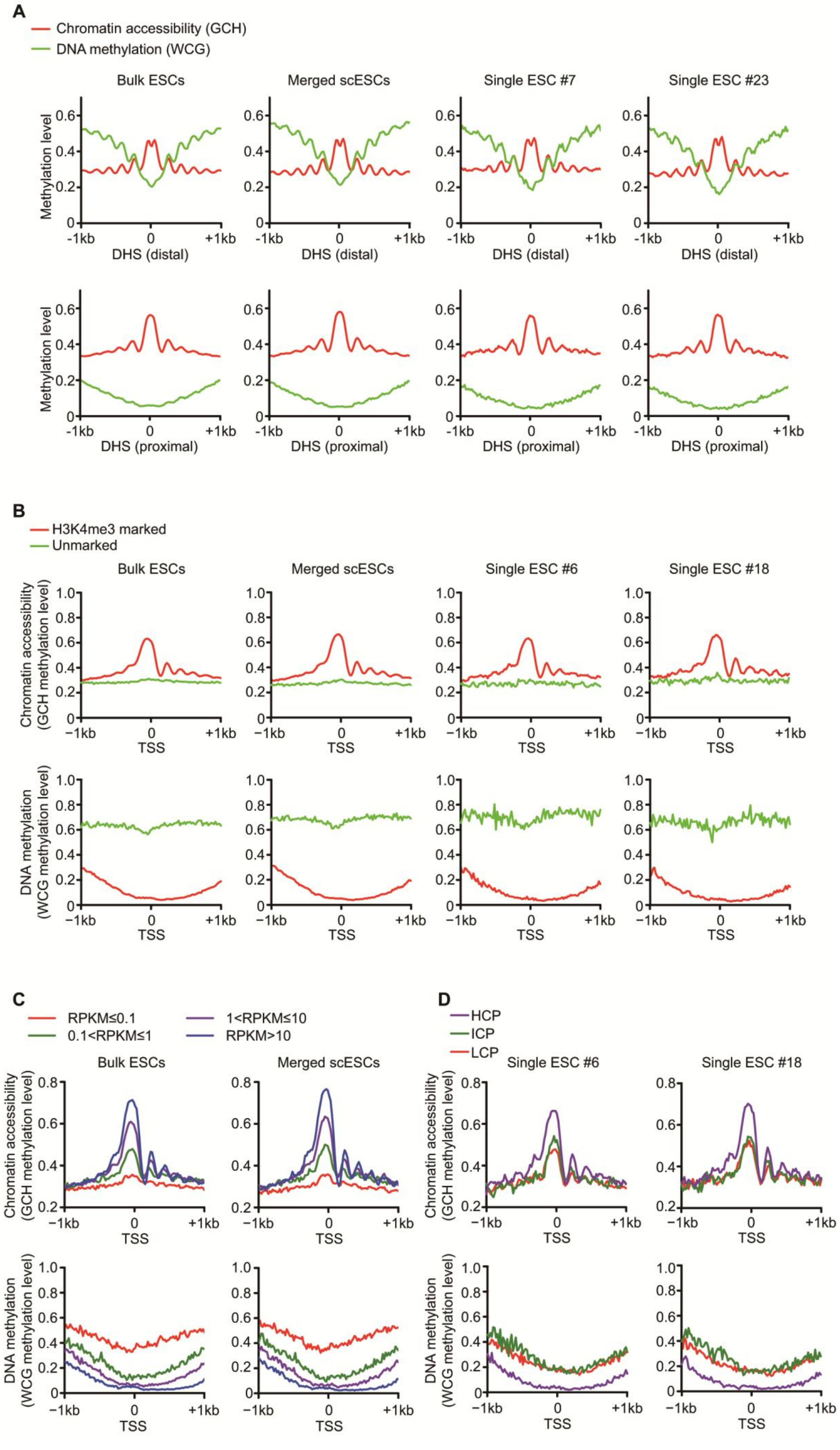

A
Chromatin accessibility (GCH)
DNA methylation (WCG)
Bulk ESCs
Merged scESCs
Single ESC #7
Single ESC #23
Methylation level
DHS (distal)
DHS (proximal)
B
H3K4me3 marked
Unmarked
Bulk ESCs
Merged scESCs
Single ESC #6
Single ESC #18
Chromatin accessibility (GCH methylation level)
DNA methylation (WCG methylation level)
TSS
C
RPKM≤0.1
0.1<RPKM≤1
1<RPKM≤10
RPKM>10
Bulk ESCs
Merged scESCs
D
HCP
ICP
LCP
Single ESC #6
Single ESC #18
-1kb
0
+1kb

Supplementary information, Figure S4. Single-cell COOL-seq analysis reveals epigenomic signatures of chromatin accessibility in mouse ES cells.

(A) Chromatin accessibility (GCH methylation level) and DNA methylation level (WCG methylation level) around DNaseI-hypersensitive sites (DHS) in individual mouse ES cells revealed by single-cell COOL-seq analysis. The DHS of mouse bulk ES cells (175,237 DHS sites in total, 150 bp long on average) from a published DNaseI-seq dataset were divided into distal (outside the region of ±2 kb of TSS) and proximal (within ±2 kb of the TSS) groups. The average GCH level (red curve) and WCG level (green curve) of bulk or single ES cells are shown around the DHS. Bulk ES cells, 24 merged individual ESCs and 2 representative single ES cells are shown.

(B) Chromatin accessibility and DNA methylation at the H3K4me3-marked and -unmarked promoter regions. The upper panel shows the chromatin accessibility of bulk or individual ES cells around the TSS. The red curve represents the average chromatin accessibility of H3K4me3-marked promoters. The green curve represents the average chromatin accessibility of the promoters without H3K4me3 enrichment. The lower panel shows the endogenous DNA methylation of bulk or individual ES cells around the TSS with or without H3K4me3 marks (red and green curves, respectively).

(C) Promoter accessibility and DNA methylation level among silenced (RPKM ≤ 0.1), low-expression (0.1 < RPKM ≤ 1), intermediate-expression (1 < RPKM ≤ 10) and high-expression (RPKM > 10) genes. The genes were divided into 4 groups according to their RPKM value from RNA-seq of bulk mouse ES cells. The upper panel shows the chromatin accessibility, and the first three nucleosomes downstream of the TSS were strongly positioned in the highly-expressed genes, which also had the highest promoter openness. The promoter DNA methylation levels are shown in the lower panel. RNA-seq data of bulk ES cells that were the same batch as the scCOOL-seq used was included here for analysis.

(D) Promoter accessibility and DNA methylation level among the HCP (high-density CpG promoter), ICP (intermediate-density CpG promoter) and LCP (low-density CpG promoter).
