# Supplementary material for: Single-cell multi-omics sequencing of mouse early embryos and embryonic stem cells
Source: Cell Res. 2017 Jun 16;27(8):967–88. doi: 10.1038/cr.2017.82 (PMC5539349; doi:10.1038/cr.2017.82)
Supplement: Supplementary information, Figure S5 — Nucleosome positioning patterns observed by single-cell COOL-seq method. [file cr201782x5.pdf]

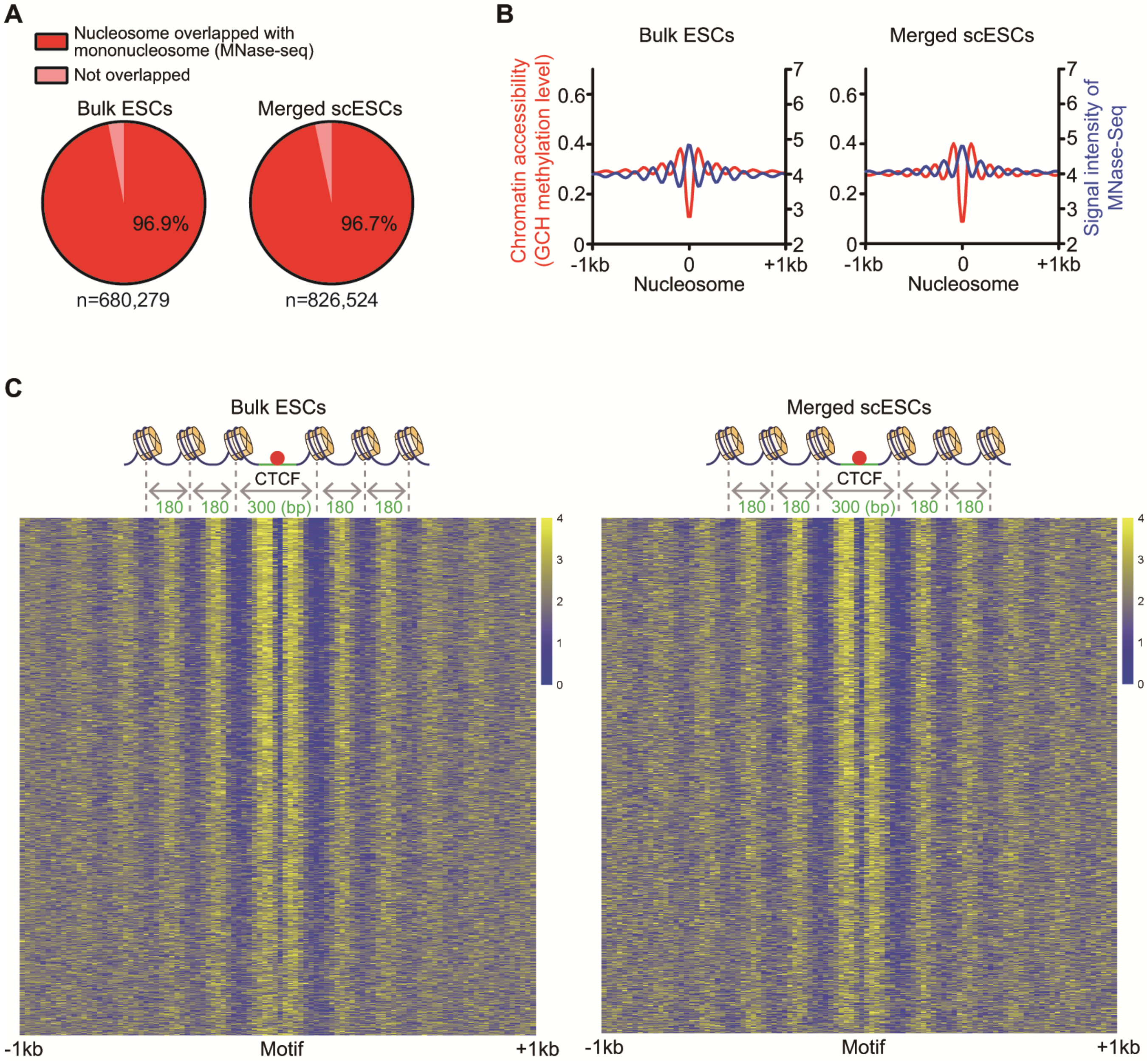
A
Nucleosome overlapped with mononucleosome (MNase-seq)
Not overlapped
Bulk ESCs
Merged scESCs
96.9%
96.7%
n=680,279
n=826,524
B
Bulk ESCs
Merged scESCs
Chromatin accessibility (GCH methylation level)
Signal intensity of MNase-Seq
-1kb
0
+1kb
Nucleosome
C
Bulk ESCs
Merged scESCs
CTCF
180
300 (bp)
-1kb
Motif
+1kb

Supplementary information, Figure S5. Nucleosome positioning patterns observed by single-cell COOL-seq method.

(A) The proportion of nucleosomes detected by single-cell COOL-seq analysis that overlapped with the mono-nucleosomes detected in bulk ES cells detected by MNase-seq.

(B) Nucleosome positioning of individual mouse ES cells revealed by single-cell COOL-seq analysis. The nucleosomes that were inaccessible to the GpC methylase were calculated using our criteria and overlapped nicely with nucleosomes detected by MNase-seq (blue curve).

(C) Heat map of nucleosome positioning around ±1 kb of the CTCF motif within the CTCF ChIP-seq peaks. Data from bulk ES cells and merged single-cell COOL-seq data from 24 ES cells were shown.
