# Supplementary material for: Single-cell multi-omics sequencing of mouse early embryos and embryonic stem cells
Source: Cell Res. 2017 Jun 16;27(8):967–88. doi: 10.1038/cr.2017.82 (PMC5539349; doi:10.1038/cr.2017.82)
Supplement: Supplementary information, Figure S6 — Representative loci detected as either open or closed chromatin by single-cell COOL-seq method were validated by liDNaseI-qPCR assay. [file cr201782x6.pdf]

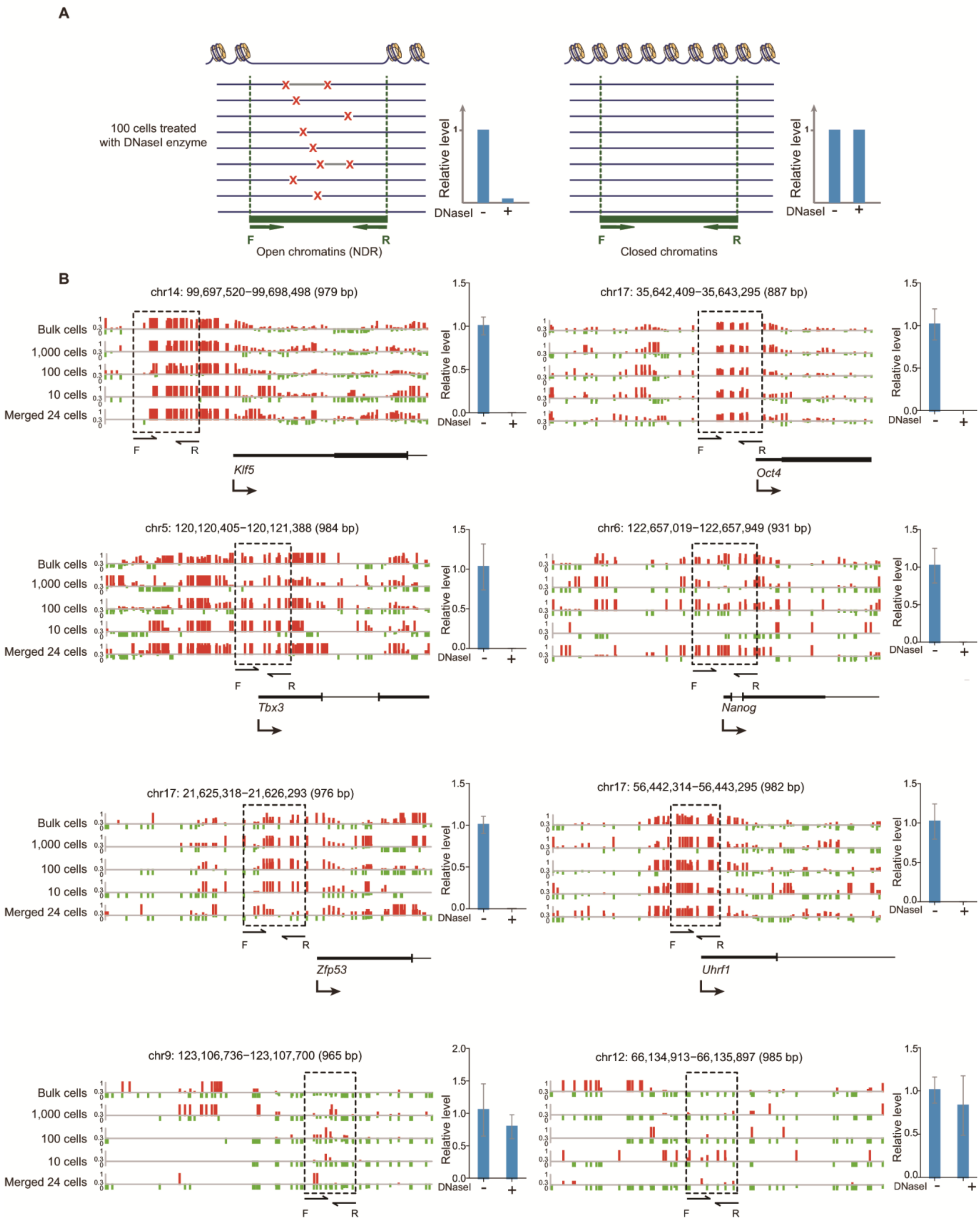

A
100 cells treated with DNaseI enzyme
Relative level
DNaseI - +
F R
Open chromatins (NDR)
Closed chromatins
B
Bulk cells
1,000 cells
100 cells
10 cells
Merged 24 cells
chr14: 99,697,520−99,698,498 (979 bp)
Klf5
chr17: 35,642,409−35,643,295 (887 bp)
Oct4
chr5: 120,120,405−120,121,388 (984 bp)
Tbx3
chr6: 122,657,019−122,657,949 (931 bp)
Nanog
chr17: 21,625,318−21,626,293 (976 bp)
Zfp53
chr17: 56,442,314−56,443,295 (982 bp)
Uhrf1
chr9: 123,106,736−123,107,700 (965 bp)
chr12: 66,134,913−66,135,897 (985 bp)

Supplementary information, Figure S6. Representative loci detected as either open or closed chromatin by single-cell COOL-seq method were validated by liDNaseI-qPCR assay.

(A) Diagram of the liDNaseI-qPCR assay. Briefly, 100 cells were treated using the protocol published recently by Yi Zhang's lab (Falong Lu *et al.*, *Cell*, 2016).

(B) Representative loci detected as open chromatins (*Klf5*, *Pou5f1*, *Tbx3*, *Nanog*, *Zfp53* and *Uhrf1*) and loci detected as closed chromatins in single-cell COOL-seq results (shown were GCH sites with averaged methylation level) were validated by liDNaseI-qPCR assay. 4 replicates were performed for each locus and the error bar indicated the standard deviation.

Reference

Lu F, Liu Y, Inoue A, Suzuki T, Zhao K, Zhang Y. Establishing chromatin regulatory landscape during mouse preimplantation development. *Cell* 2016; **165**:1375-1388.
