# Supplementary material for: Single-cell multi-omics sequencing of mouse early embryos and embryonic stem cells
Source: Cell Res. 2017 Jun 16;27(8):967–88. doi: 10.1038/cr.2017.82 (PMC5539349; doi:10.1038/cr.2017.82)
Supplement: Supplementary information, Figure S7 — Robust and accurate detection of NDRs and nucleosomes across individual ES cells. [file cr201782x7.pdf]

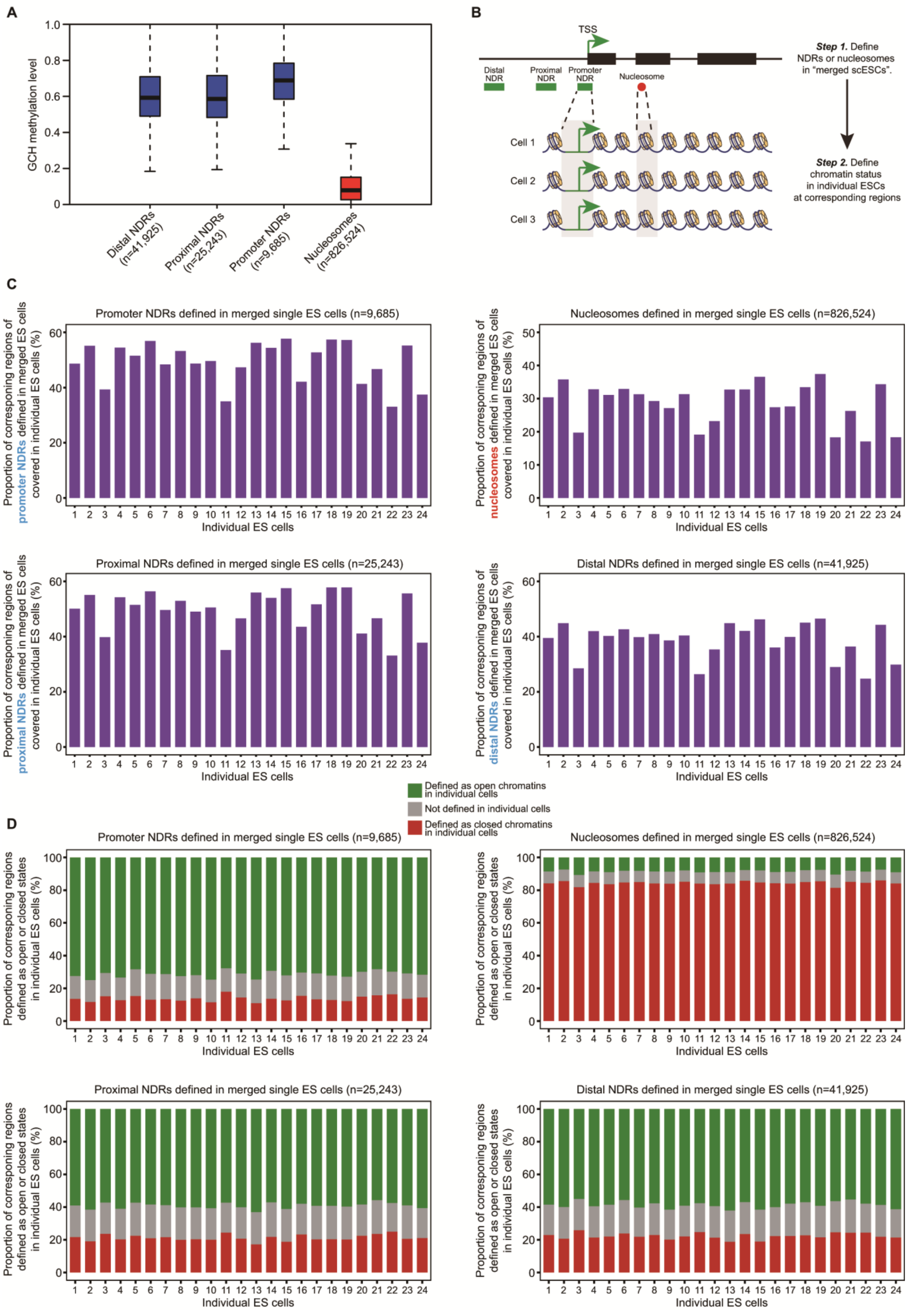
A
GCH methylation level
Distal NDRs (n=41,925)
Proximal NDRs (n=25,243)
Promoter NDRs (n=9,685)
Nucleosomes (n=826,524)
B
TSS
Distal NDR
Proximal NDR
Promoter NDR
Nucleosome
Cell 1
Cell 2
Cell 3
Step 1. Define NDRs or nucleosomes in "merged scESCs".
Step 2. Define chromatin status in individual ESCs at corresponding regions
C
Promoter NDRs defined in merged single ES cells (n=9,685)
Nucleosomes defined in merged single ES cells (n=826,524)
Proximal NDRs defined in merged single ES cells (n=25,243)
Distal NDRs defined in merged single ES cells (n=41,925)
Proportion of corresponing regions of promoter NDRs defined in merged ES cells covered in individual ES cells (%)
Proportion of corresponing regions of nucleosomes defined in merged ES cells covered in individual ES cells (%)
Proportion of corresponing regions of proximal NDRs defined in merged ES cells covered in individual ES cells (%)
Proportion of corresponing regions of distal NDRs defined in merged ES cells covered in individual ES cells (%)
Individual ES cells
Defined as open chromatins in individual cells
Not defined in individual cells
Defined as closed chromatins in individual cells
D
Promoter NDRs defined in merged single ES cells (n=9,685)
Nucleosomes defined in merged single ES cells (n=826,524)
Proximal NDRs defined in merged single ES cells (n=25,243)
Distal NDRs defined in merged single ES cells (n=41,925)
Proportion of corresponing regions defined as open or closed states in individual ES cells (%)
Individual ES cells

Supplementary information, Figure S7. Robust and accurate detection of NDRs and nucleosomes across individual ES cells.

(A) Boxplot of averaged GCH methylation level of NDRs (distal, proximal and promoter NDRs) and nucleosomes defined in merged single ES cells (promoter NDRs, NDRs contain TSS; proximal NDRs, NDRs within 2kb upstream and downstream of the TSS; distal NDRs, NDRs at least 2kb away from the TSS).

(B) Diagram of definition of chromatin status across individual ES cells. if an NDR or nucleosome defined in merged single cell samples is covered in a single cell (at least 5 GCH sites covered in corresponding regions) with the averaged GCH methylation level above 0.5, this region is defined as open chromatin in this single cell; If an NDR or nucleosome defined in merged single cell samples is covered in a single cell with the averaged GCH methylation level of 0.3, this region is defined as closed chromatin in this single cell.

(C) Proportion of the corresponding regions that defined as NDRs or nucleosomes in merged single ES cells covered in individual ES cells.

(D) Proportion of corresponding regions defined as open or closed in individual ES cells.
