# Supplementary material for: Single-cell multi-omics sequencing of mouse early embryos and embryonic stem cells
Source: Cell Res. 2017 Jun 16;27(8):967–88. doi: 10.1038/cr.2017.82 (PMC5539349; doi:10.1038/cr.2017.82)
Supplement: Supplementary information, Figure S8 — Variation of DNA methylation and chromatin accessibility at specific genomic elements among different individual cells at each developmental stage. [file cr201782x8.pdf]

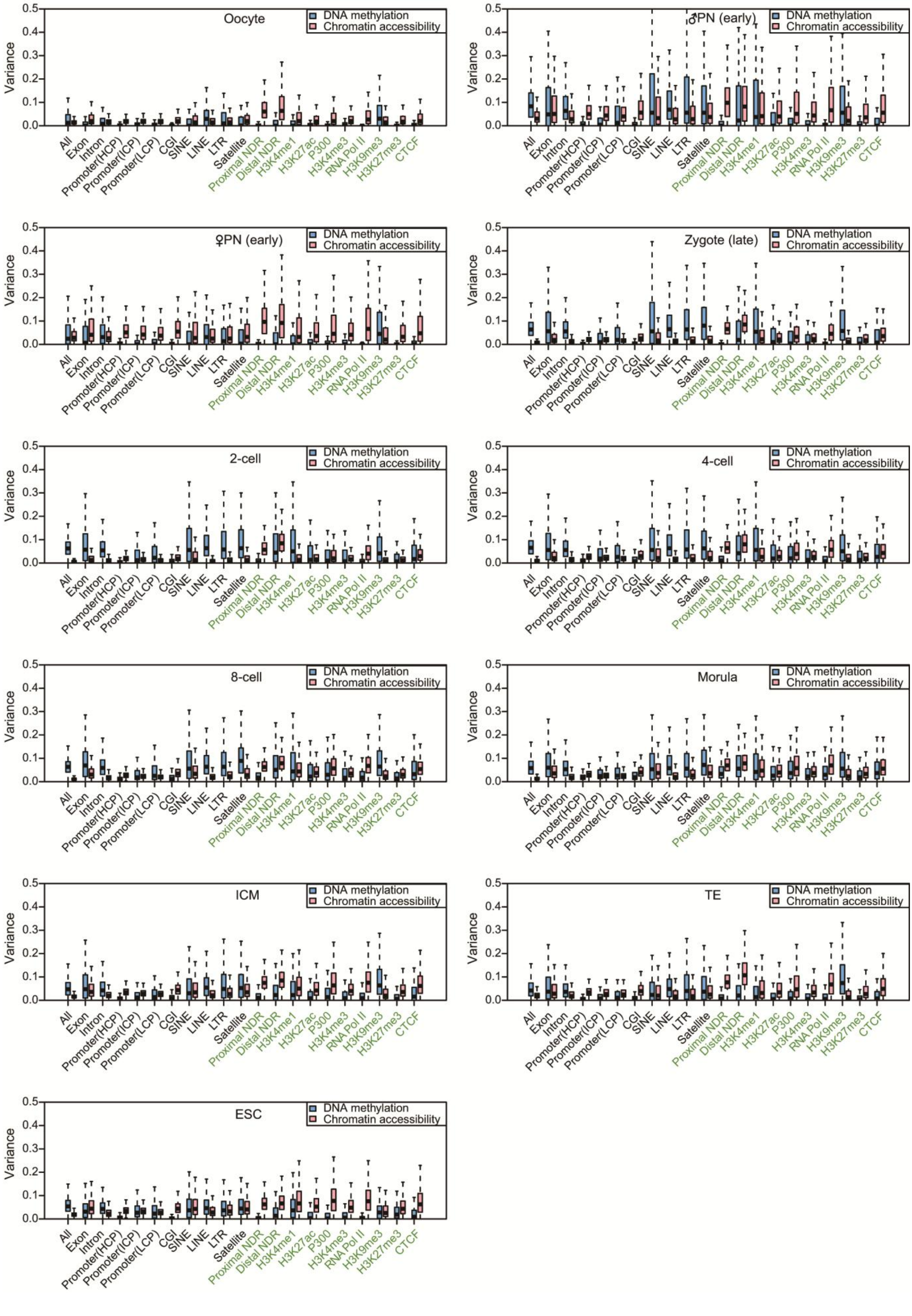

Oocyte
♂PN (early)
♀PN (early)
Zygote (late)
2-cell
4-cell
8-cell
Morula
ICM
TE
ESC
DNA methylation
Chromatin accessibility
Variance
0.0
0.1
0.2
0.3
0.4
0.5
All
Exon
Intron
Promoter(HCP)
Promoter(ICP)
Promoter(LCP)
CGI
SINE
LINE
LTR
Satellite
Proximal NDR
Distal NDR
H3K4me1
H3K27ac
P300
H3K4me3
RNA Pol II
H3K9me3
H3K27me3
CTCF

Supplementary information, Figure S8. Variation of DNA methylation and chromatin accessibility at specific genomic elements among different individual cells at each developmental stage. Variation of DNA methylation and chromatin accessibility at exon, intron, promoters, CGIs and repetitive elements in each developmental stage. Variation of DNA methylation and chromatin accessibility at putative regulatory elements. NDRs that detected within each stage were used. The published ChIP-seq data were from mouse ES cells.
