# Supplementary material for: Single-cell multi-omics sequencing of mouse early embryos and embryonic stem cells
Source: Cell Res. 2017 Jun 16;27(8):967–88. doi: 10.1038/cr.2017.82 (PMC5539349; doi:10.1038/cr.2017.82)
Supplement: Supplementary information, Figure S9 — Chromatin accessibility of mouse preimplantation embryos revealed by single-cell COOL-seq analysis. [file cr201782x9.pdf]

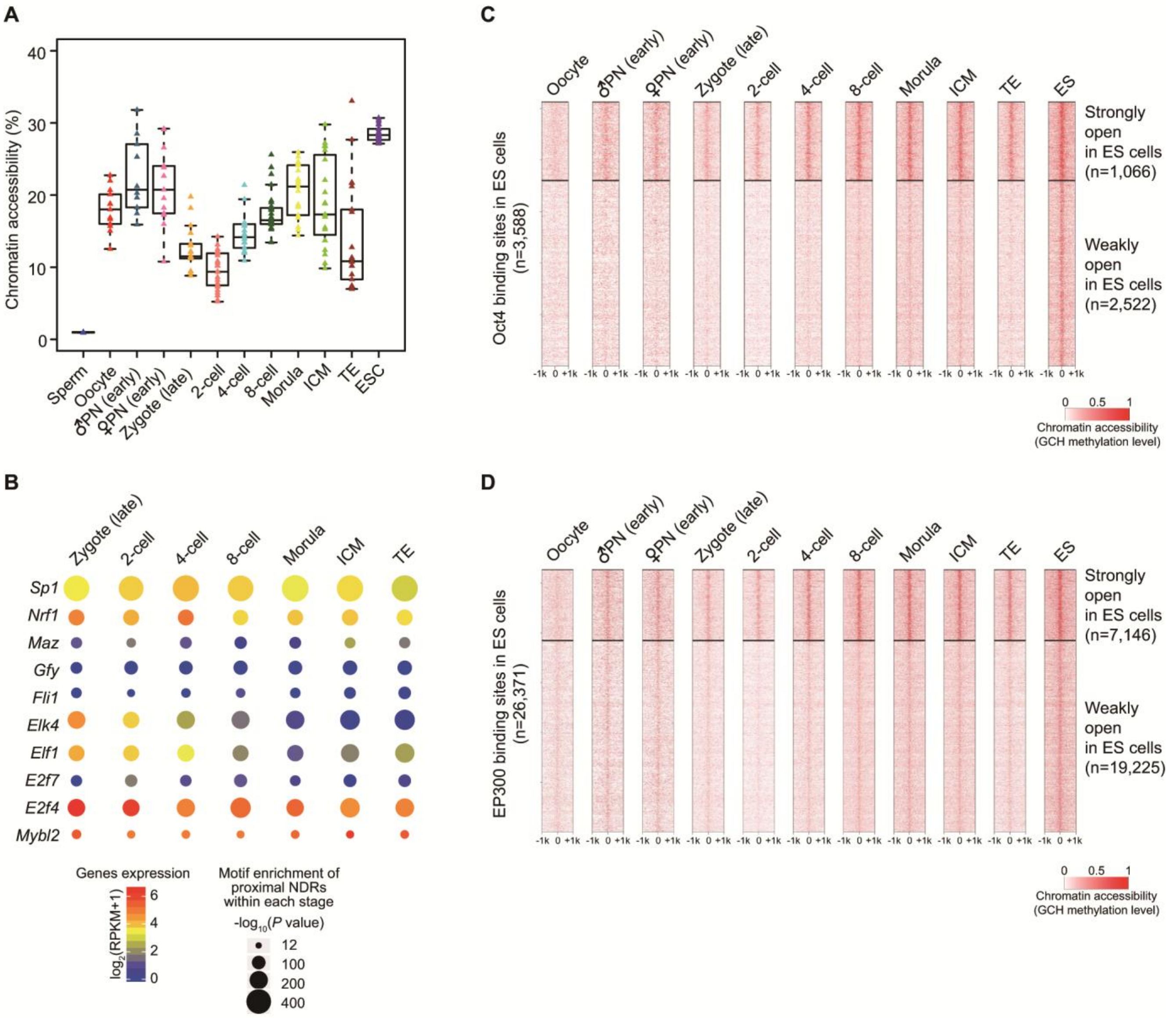
A
Chromatin accessibility (%)
Sperm
Oocyte
♂PN (early)
♀PN (early)
Zygote (late)
2-cell
4-cell
8-cell
Morula
ICM
TE
ESC
B
Sp1
Nrf1
Maz
Gfy
Fli1
Elk4
Elf1
E2f7
E2f4
Mybl2
Genes expression
log2(RPKM+1)
Motif enrichment of proximal NDRs within each stage
-log10(P value)
C
Oct4 binding sites in ES cells (n=3,588)
Strongly open in ES cells (n=1,066)
Weakly open in ES cells (n=2,522)
Chromatin accessibility (GCH methylation level)
D
EP300 binding sites in ES cells (n=26,371)
Strongly open in ES cells (n=7,146)
Weakly open in ES cells (n=19,225)
Chromatin accessibility (GCH methylation level)

Supplementary information, Figure S9. Chromatin accessibility of mouse preimplantation embryos revealed by single-cell COOL-seq analysis.

(A) Boxplot of averaged chromatin accessibility in each single blastomere. The mean methylation level of all the detected GCH sites in a single cell was calculated. The cells at the same developmental stage were plotted together in the same box.

(B) Representative motifs with their P-value of motif enrichment analysis of nucleosome depleted regions around TSS during mouse preimplantation development.

(C) Dynamic of chromatin accessibility of presumable binding sites of Oct4 during preimplantation development. 3,588 of Oct4 binding sites identified in mouse ES cells by ChIP-seq were used for analysis. Those binding sites covered (at least 5 GCH sites covered in corresponding regions) across all the stages were used for analysis.

(D) Dynamic of chromatin accessibility of presumable binding sites of EP300 during preimplantation development. 26,371 of EP300 binding sites identified in mouse ES cells by ChIP-seq were used for analysis. Those binding sites covered (at least 5 GCH sites covered in corresponding regions) across all the stages were used for analysis.
