# Supplementary material for: Single-cell multi-omics sequencing of mouse early embryos and embryonic stem cells
Source: Cell Res. 2017 Jun 16;27(8):967–88. doi: 10.1038/cr.2017.82 (PMC5539349; doi:10.1038/cr.2017.82)
Supplement: Supplementary information, Figure S10 — Chromatin accessibility and DNA methylation at promoters, NDRs and nucleosomes during preimplantation development. [file cr201782x10.pdf]

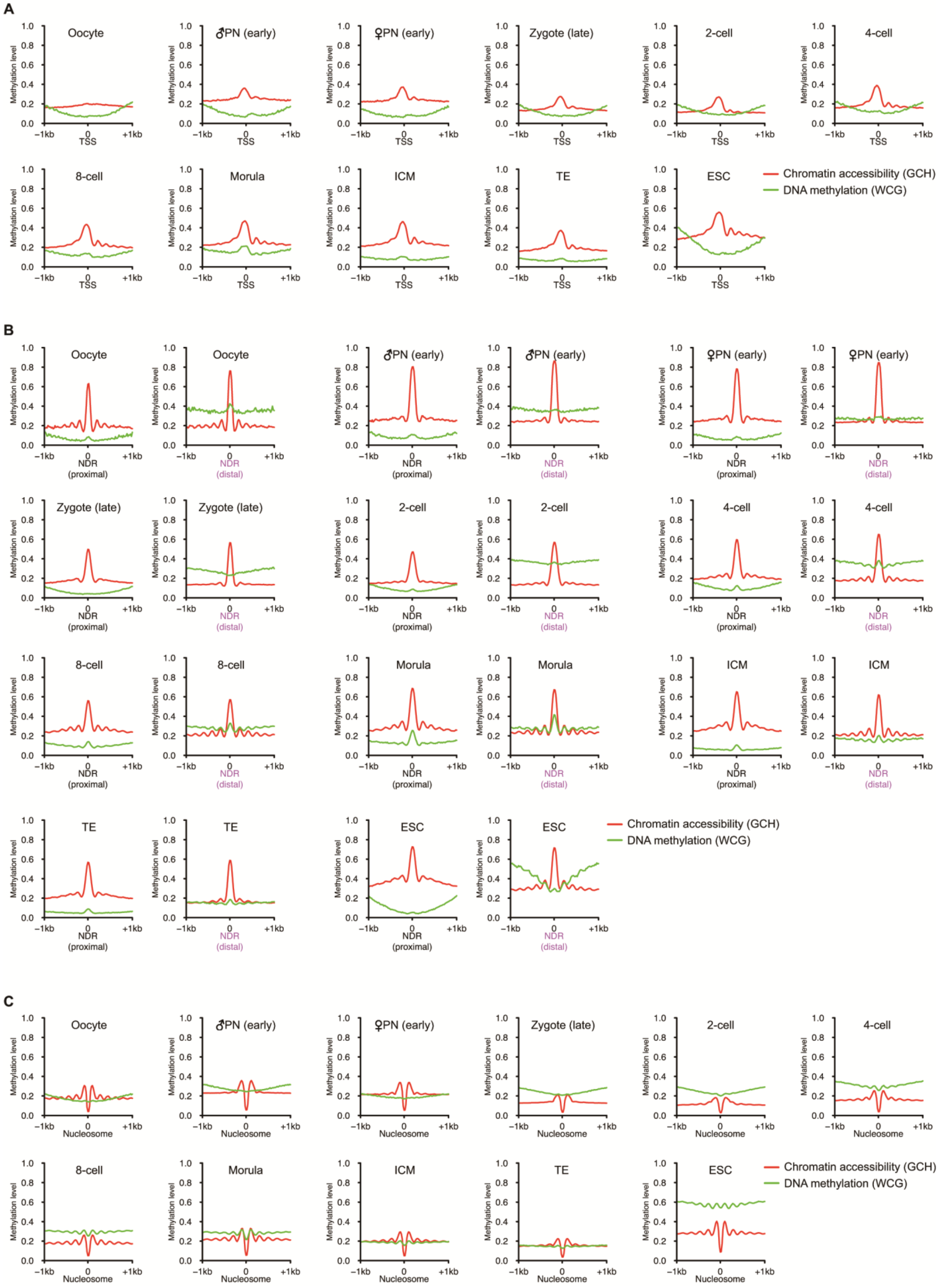
A
Oocyte
♂PN (early)
♀PN (early)
Zygote (late)
2-cell
4-cell
8-cell
Morula
ICM
TE
ESC
Methylation level
TSS
−1kb
+1kb
Chromatin accessibility (GCH)
DNA methylation (WCG)
B
NDR
(proximal)
(distal)
C
Nucleosome

Supplementary information, Figure S10. Chromatin accessibility and DNA methylation at promoters, NDRs and nucleosomes during preimplantation development.

(A) Chromatin accessibility and DNA methylation at the promoter regions. The red curve represents the average chromatin accessibility of the promoters. The green curve shows the endogenous DNA methylation within each stage.

(B) Chromatin accessibility and DNA methylation at the proximal and distal NDRs within each stage.

(C) Chromatin accessibility and DNA methylation at the nucleosomes within each stage.
