# Supplementary material for: Single-cell multi-omics sequencing of mouse early embryos and embryonic stem cells
Source: Cell Res. 2017 Jun 16;27(8):967–88. doi: 10.1038/cr.2017.82 (PMC5539349; doi:10.1038/cr.2017.82)
Supplement: Supplementary information, Figure S11 — Dynamics of chromatin accessibility of different functional genomic elements in mouse early embryos. [file cr201782x11.pdf]

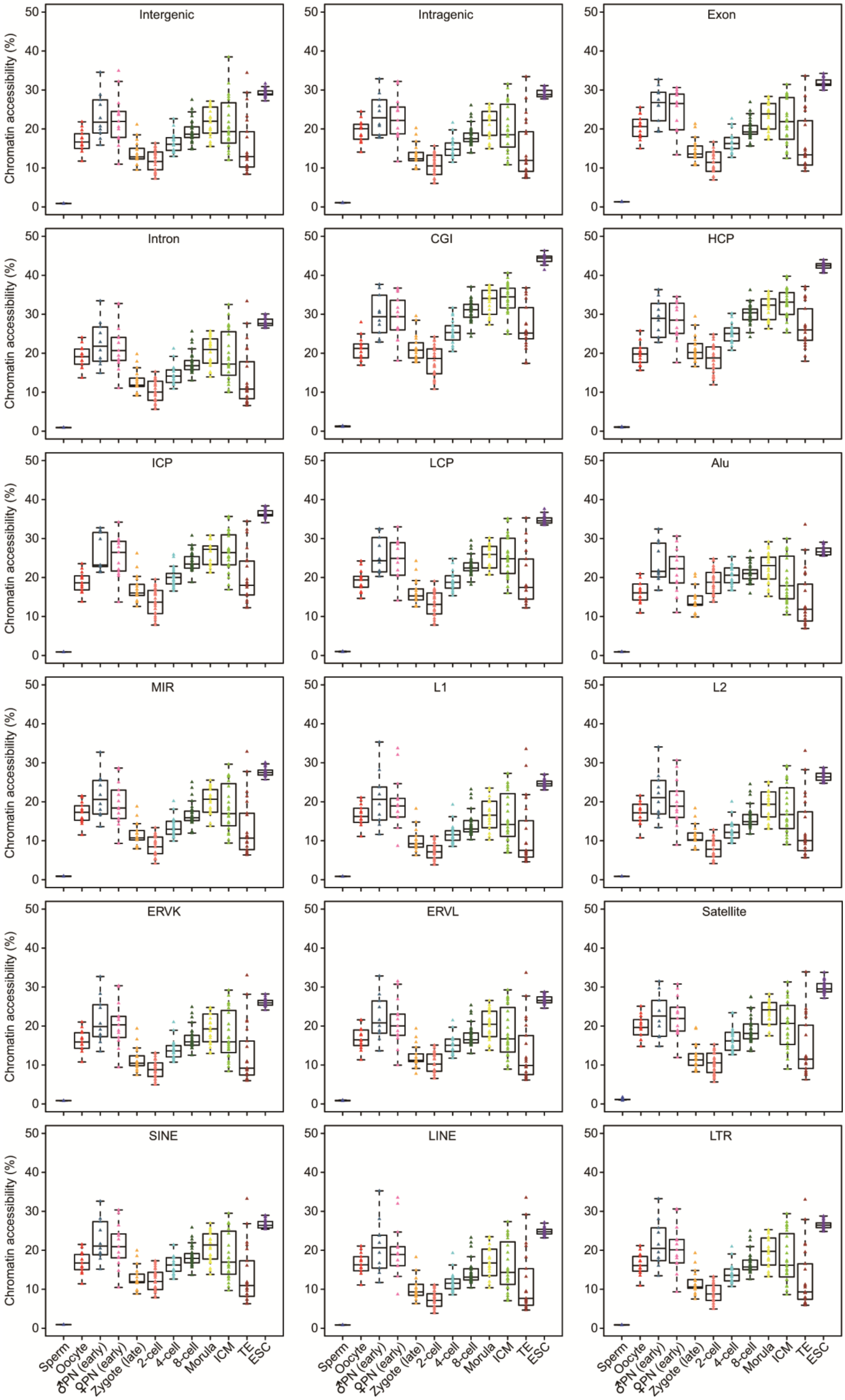
Intergenic
Intragenic
Exon
Intron
CGI
HCP
ICP
LCP
Alu
MIR
L1
L2
ERVK
ERVL
Satellite
SINE
LINE
LTR
Chromatin accessibility (%)
0
10
20
30
40
50
Sperm
Oocyte
♂PN (early)
♀PN (early)
Zygote (late)
2-cell
4-cell
8-cell
Morula
ICM
TE
ESC

Supplementary information, Figure S11. Dynamics of chromatin accessibility of different functional genomic elements in mouse early embryos. Boxplots of chromatin accessibility in different genomic regions, including intergenic regions, intragenic regions, exons, introns, CGIs, promoters and subgroups of repetitive elements. The bottom and top of the boxes indicate the first and third quartiles, respectively, and the lines inside the boxes indicate the medians of the data. Each triangle indicates a single cell or blastomere. Note that the sperm samples were 9 bulk cell samples.
