# Supplementary material for: Single-cell multi-omics sequencing of mouse early embryos and embryonic stem cells
Source: Cell Res. 2017 Jun 16;27(8):967–88. doi: 10.1038/cr.2017.82 (PMC5539349; doi:10.1038/cr.2017.82)
Supplement: Supplementary information, Figure S12 — Dynamics of chromatin accessibility of subfamilies of SINEs. [file cr201782x12.pdf]

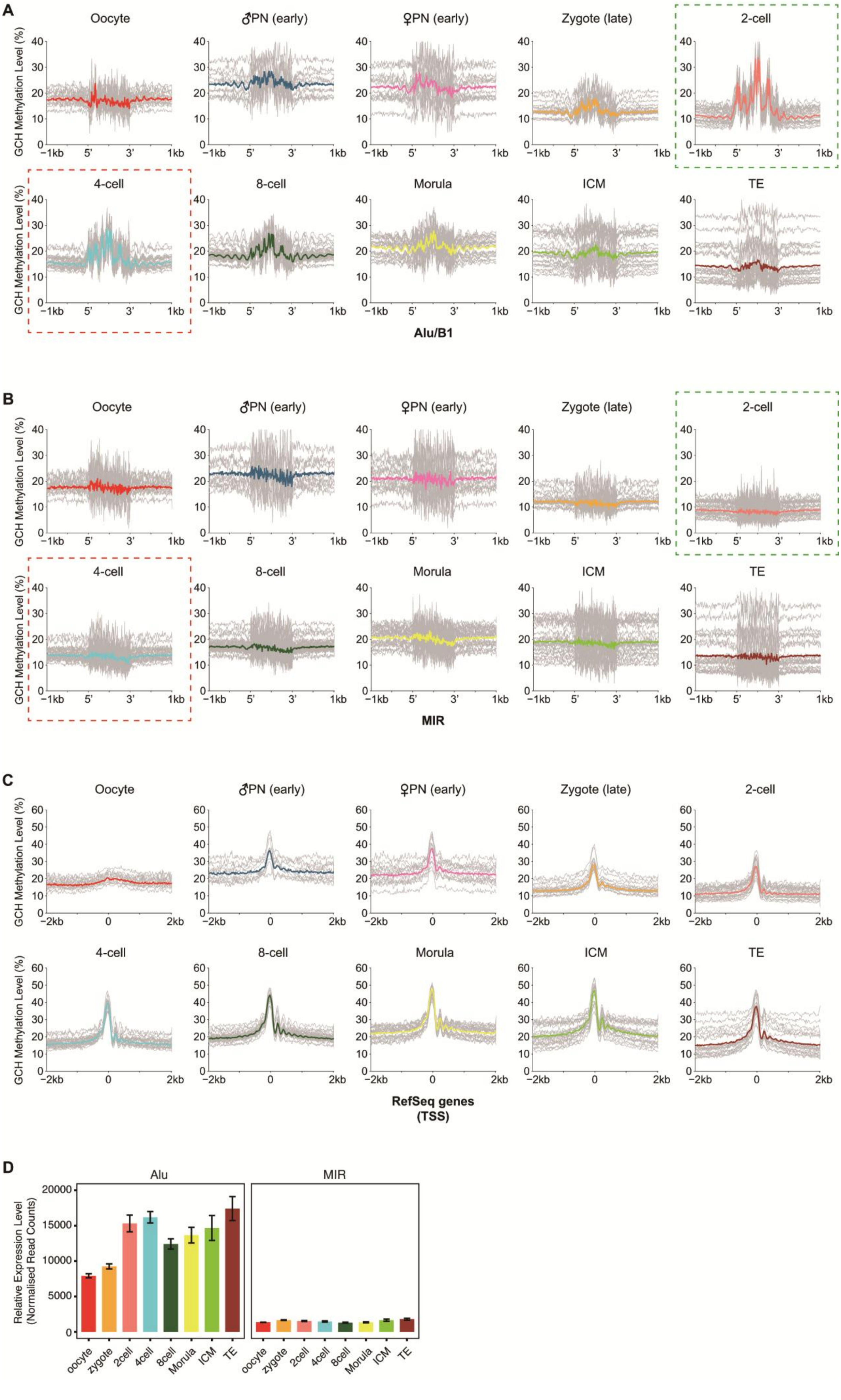

A
Oocyte
♂PN (early)
♀PN (early)
Zygote (late)
2-cell
4-cell
8-cell
Morula
ICM
TE
GCH Methylation Level (%)
0
10
20
30
40
−1kb
5'
3'
1kb
Alu/B1
B
Oocyte
♂PN (early)
♀PN (early)
Zygote (late)
2-cell
4-cell
8-cell
Morula
ICM
TE
GCH Methylation Level (%)
−1kb
5'
3'
1kb
MIR
C
Oocyte
♂PN (early)
♀PN (early)
Zygote (late)
2-cell
4-cell
8-cell
Morula
ICM
TE
GCH Methylation Level (%)
0
10
20
30
40
50
60
−2kb
0
2kb
RefSeq genes
(TSS)
D
Alu
MIR
Relative Expression Level
(Normalised Read Counts)
0
5000
10000
15000
20000
oocyte
zygote
2cell
4cell
8cell
Morula
ICM
TE

Supplementary information, Figure S12. Dynamics of chromatin accessibility of subfamilies of SINEs.

(A) The chromatin accessibility along the gene bodies, 1 kb upstream of the TSS and 1 kb downstream of the transcription end sites (TES) of Alu/B1 among individual cells across different developmental stages. The grey line indicated the GCH methylation level of each single cell. The color line indicated the GCH methylation level of merged single cells within each stage.

(B) The chromatin accessibility along the gene bodies, 1 kb upstream of the TSS and 1 kb downstream of the TES of MIR among individual cells across different developmental stages.

(C) The chromatin accessibility around TSS among individual cells across all the preimplantation stages.

(D) RNA expression of Alu/B1 and MIR across preimplantation developmental stages.
