# Supplementary material for: Single-cell multi-omics sequencing of mouse early embryos and embryonic stem cells
Source: Cell Res. 2017 Jun 16;27(8):967–88. doi: 10.1038/cr.2017.82 (PMC5539349; doi:10.1038/cr.2017.82)
Supplement: Supplementary information, Figure S13 — Dynamic of DNA methylation and chromatin accessibility of parental genomes within individual cells in preimplantation embryos. [file cr201782x13.pdf]

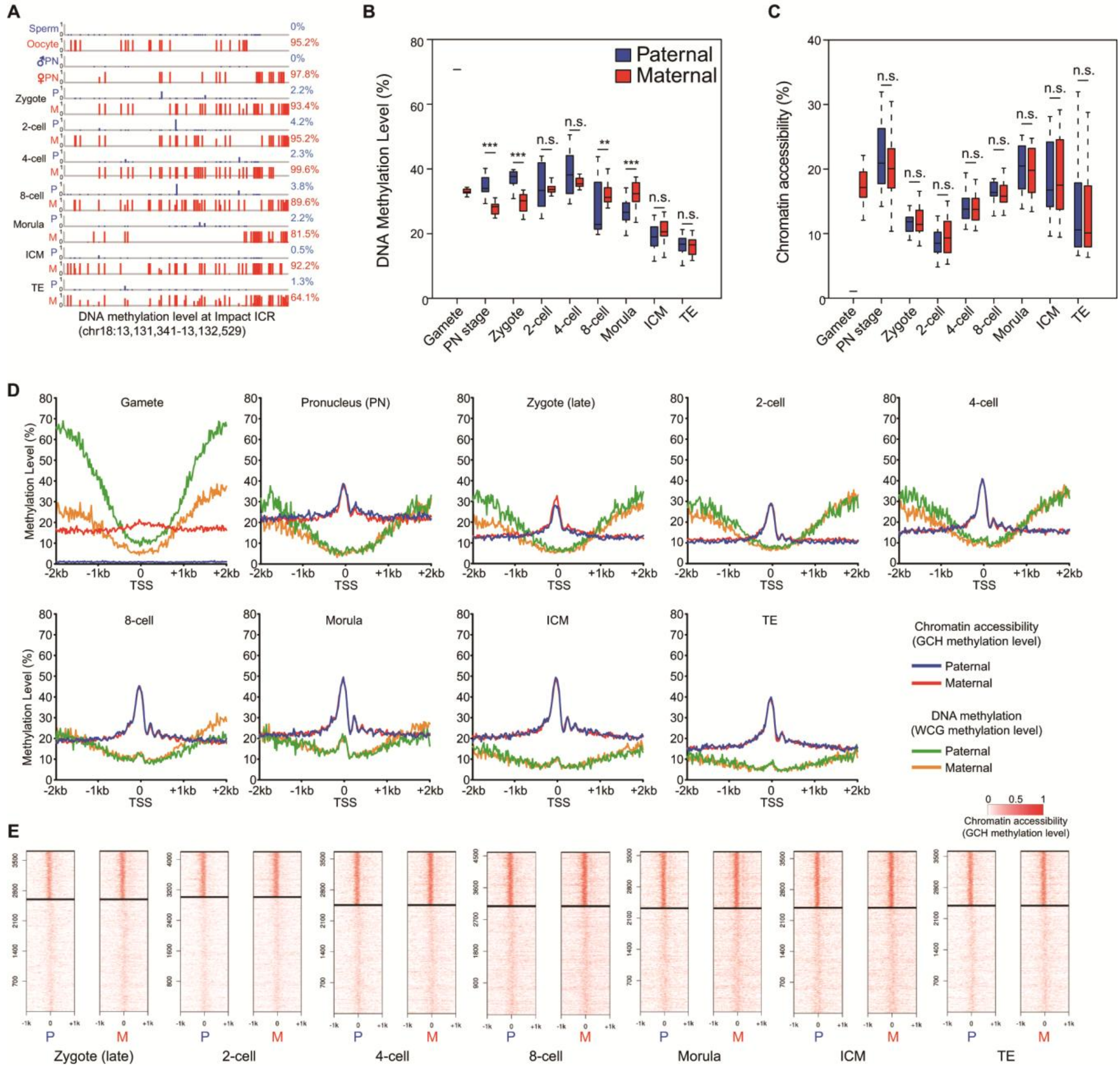

A
Sperm
Oocyte
♂PN
♀PN
Zygote
2-cell
4-cell
8-cell
Morula
ICM
TE
0%
95.2%
0%
97.8%
2.2%
93.4%
4.2%
95.2%
2.3%
99.6%
3.8%
89.6%
2.2%
81.5%
0.5%
92.2%
1.3%
64.1%
DNA methylation level at Impact ICR
(chr18:13,131,341-13,132,529)
B
DNA Methylation Level (%)
Paternal
Maternal
Gamete
PN stage
Zygote
2-cell
4-cell
8-cell
Morula
ICM
TE
***
**
n.s.
C
Chromatin accessibility (%)
D
Gamete
Pronucleus (PN)
Zygote (late)
2-cell
4-cell
8-cell
Morula
ICM
TE
Methylation Level (%)
TSS
Chromatin accessibility
(GCH methylation level)
Paternal
Maternal
DNA methylation
(WCG methylation level)
Paternal
Maternal
E
Chromatin accessibility
(GCH methylation level)
P
M
Zygote (late)
2-cell
4-cell
8-cell
Morula
ICM
TE

Supplementary information, Figure S13. Dynamic of DNA methylation and chromatin accessibility of parental genomes within individual cells in preimplantation embryos.

(A) Confirmation of the accuracy of our SNP tracing and DNA methylation detection of parental genomes by analyzing the DNA methylation status of a maternal imprinting control region (*Impact*).

(B) Boxplot of DNA methylation level of parental DNA within individual cells across preimplantation development.

(C) Boxplot of chromatin accessibility of parental DNA within individual cells across preimplantation development.

(D) Chromatin accessibility and DNA methylation level of parental genomes around the TSS across preimplantation development. Maternal and paternal genomes (WCG sites or GCH sites) traced from the merged single blastomeres within the same developmental stages were used.

(E) Heat map based on K-means clustering of promoter accessibility of both maternal and paternal genomes across preimplantation development. Maternal and paternal genomes (WCG sites or GCH sites) traced from the merged single blastomeres within the same developmental stages were used. The scale indicated number of genes analyzed within each stage.
