# Supplementary material for: Single-cell multi-omics sequencing of mouse early embryos and embryonic stem cells
Source: Cell Res. 2017 Jun 16;27(8):967–88. doi: 10.1038/cr.2017.82 (PMC5539349; doi:10.1038/cr.2017.82)
Supplement: Supplementary information, Figure S14 — Heterogeneity analysis of promoter accessibility in preimplantation embryos. [file cr201782x14.pdf]

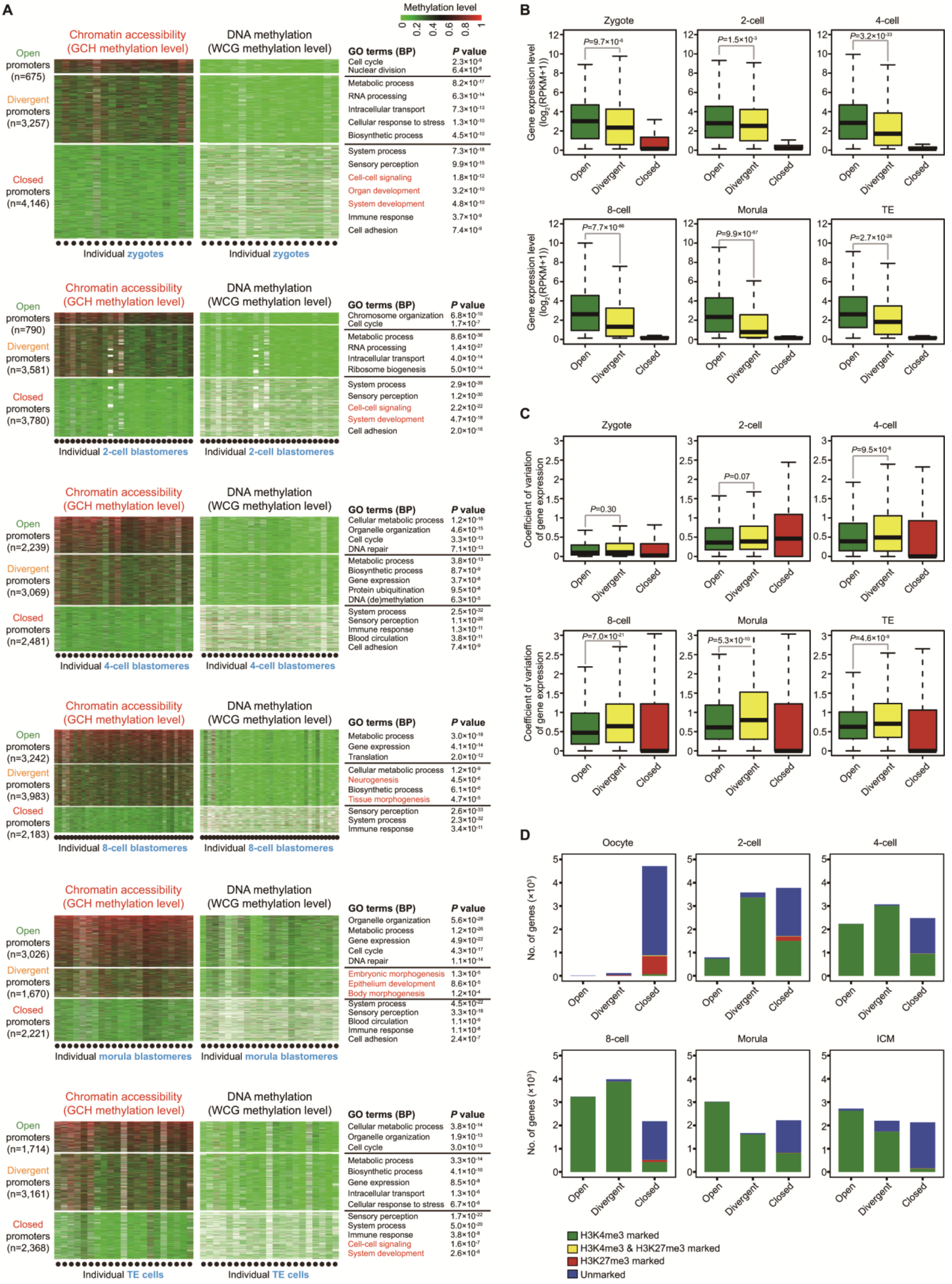

A
Methylation level
0 0.2 0.4 0.6 0.8 1
Chromatin accessibility (GCH methylation level)
DNA methylation (WCG methylation level)
Open promoters (n=675)
Divergent promoters (n=3,257)
Closed promoters (n=4,146)
GO terms (BP) P value
Cell cycle 2.3×10-9
Nuclear division 6.4×10-8
Metabolic process 8.2×10-17
RNA processing 6.3×10-14
Intracellular transport 7.3×10-13
Cellular response to stress 1.3×10-10
Biosynthetic process 4.5×10-10
System process 7.3×10-18
Sensory perception 9.9×10-15
Cell-cell signaling 1.8×10-12
Organ development 3.2×10-10
System development 4.8×10-10
Immune response 3.7×10-9
Cell adhesion 7.4×10-9
Individual zygotes
Open promoters (n=790)
Divergent promoters (n=3,581)
Closed promoters (n=3,780)
Chromosome organization 6.8×10-10
Cell cycle 1.7×10-7
Metabolic process 8.6×10-36
RNA processing 1.4×10-27
Intracellular transport 4.0×10-14
Ribosome biogenesis 5.0×10-14
System process 2.9×10-39
Sensory perception 1.2×10-30
Cell-cell signaling 2.2×10-22
System development 4.7×10-18
Cell adhesion 2.0×10-16
Individual 2-cell blastomeres
Open promoters (n=2,239)
Divergent promoters (n=3,069)
Closed promoters (n=2,481)
Cellular metabolic process 1.2×10-16
Organelle organization 4.6×10-15
Cell cycle 3.3×10-13
DNA repair 7.1×10-13
Metabolic process 3.8×10-13
Biosynthetic process 8.7×10-9
Gene expression 3.7×10-8
Protein ubiquitination 9.5×10-8
DNA (de)methylation 6.3×10-5
System process 2.5×10-32
Sensory perception 1.1×10-26
Immune response 1.3×10-11
Blood circulation 3.8×10-11
Cell adhesion 7.4×10-9
Individual 4-cell blastomeres
Open promoters (n=3,242)
Divergent promoters (n=3,983)
Closed promoters (n=2,183)
Metabolic process 3.0×10-18
Gene expression 4.1×10-14
Translation 2.0×10-12
Cellular metabolic process 1.2×10-9
Neurogenesis 4.5×10-6
Biosynthetic process 6.1×10-6
Tissue morphogenesis 4.7×10-5
Sensory perception 2.6×10-33
System process 2.3×10-32
Immune response 3.4×10-11
Individual 8-cell blastomeres
Open promoters (n=3,026)
Divergent promoters (n=1,670)
Closed promoters (n=2,221)
Organelle organization 5.6×10-28
Metabolic process 1.2×10-26
Gene expression 4.9×10-22
Cell cycle 4.3×10-17
DNA repair 1.1×10-14
Embryonic morphogenesis 1.3×10-5
Epithelium development 8.6×10-5
Body morphogenesis 1.2×10-4
System process 4.5×10-22
Sensory perception 3.3×10-18
Blood circulation 1.1×10-9
Immune response 1.1×10-8
Cell adhesion 2.4×10-7
Individual morula blastomeres
Open promoters (n=1,714)
Divergent promoters (n=3,161)
Closed promoters (n=2,368)
Cellular metabolic process 3.8×10-14
Organelle organization 1.9×10-13
Cell cycle 3.0×10-13
Metabolic process 3.3×10-14
Biosynthetic process 4.1×10-10
Gene expression 8.5×10-8
Intracellular transport 1.3×10-6
Cellular response to stress 6.7×10-6
Sensory perception 1.7×10-22
System process 5.0×10-20
Immune response 3.8×10-8
Cell-cell signaling 1.6×10-7
System development 2.6×10-6
Individual TE cells
B
Zygote
2-cell
4-cell
8-cell
Morula
TE
Gene expression level (log2(RPKM+1))
P=9.7×10-6
P=1.5×10-3
P=3.2×10-33
P=7.7×10-66
P=9.9×10-67
P=2.7×10-26
Open
Divergent
Closed
C
Coefficient of variation of gene expression
P=0.30
P=0.07
P=9.5×10-8
P=7.0×10-21
P=5.3×10-10
P=4.6×10-9
D
Oocyte
2-cell
4-cell
8-cell
Morula
ICM
No. of genes (×10³)
H3K4me3 marked
H3K4me3 & H3K27me3 marked
H3K27me3 marked
Unmarked

Supplementary information, Figure S14. Heterogeneity analysis of promoter accessibility in preimplantation embryos.

(A) Chromatin accessibility and DNA methylation level of homogeneously open promoters, homogeneously closed promoters and divergent promoters of individual blastomeres within each developmental stage.

(B) Gene expression of corresponding genes with homogeneously open promoters, homogeneously closed promoters and divergent promoters in mouse preimplantation embryos.

(C) Coefficient of variation (CV) of expression levels of the corresponding genes with homogeneously open promoters, homogeneously closed promoters and divergent promoters among individual blastomeres in mouse preimplantation embryos.

(D) The number of genes within each category that had either H3K4me3 or H3K27me3 marks in mouse preimplantation embryos. ChIP-seq Data was from previous publication (Xiaoyu Liu *et al.*, *Nature*. 2016).

Reference

Liu X, Wang C, Liu W *et al.* Distinct features of H3K4me3 and H3K27me3 chromatin domains in pre-implantation embryos. *Nature* 2016; **537**:558-562.
