# Supplementary material for: Single-cell multi-omics sequencing of mouse early embryos and embryonic stem cells
Source: Cell Res. 2017 Jun 16;27(8):967–88. doi: 10.1038/cr.2017.82 (PMC5539349; doi:10.1038/cr.2017.82)
Supplement: Supplementary information, Figure S15 — The relationship among DNA methylation, chromatin accessibility and expression of RefSeq genes during mouse preimplantation development. [file cr201782x15.pdf]

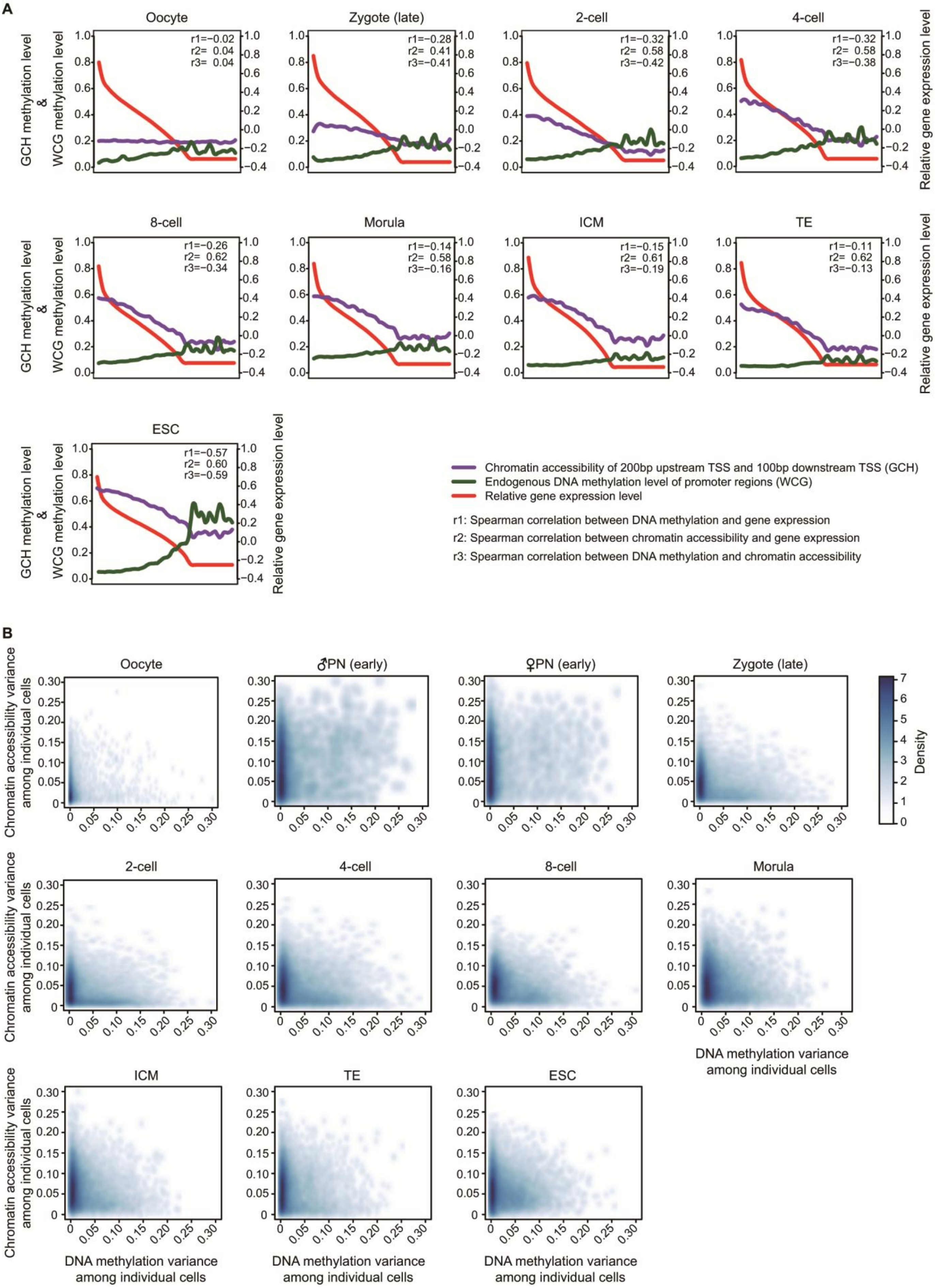
A
Oocyte
Zygote (late)
2-cell
4-cell
8-cell
Morula
ICM
TE
ESC
r1=−0.02
r2= 0.04
r3= 0.04
r1=−0.28
r2= 0.41
r3=−0.41
r1=−0.32
r2= 0.58
r3=−0.42
r1=−0.32
r2= 0.58
r3=−0.38
r1=−0.26
r2= 0.62
r3=−0.34
r1=−0.14
r2= 0.58
r3=−0.16
r1=−0.15
r2= 0.61
r3=−0.19
r1=−0.11
r2= 0.62
r3=−0.13
r1=−0.57
r2= 0.60
r3=−0.59
GCH methylation level & WCG methylation level
Relative gene expression level
Chromatin accessibility of 200bp upstream TSS and 100bp downstream TSS (GCH)
Endogenous DNA methylation level of promoter regions (WCG)
Relative gene expression level
r1: Spearman correlation between DNA methylation and gene expression
r2: Spearman correlation between chromatin accessibility and gene expression
r3: Spearman correlation between DNA methylation and chromatin accessibility
B
Oocyte
♂PN (early)
♀PN (early)
Zygote (late)
2-cell
4-cell
8-cell
Morula
ICM
TE
ESC
Density
Chromatin accessibility variance among individual cells
DNA methylation variance among individual cells

Supplementary information, Figure S15. The relationship among DNA methylation, chromatin accessibility and expression of RefSeq genes during mouse preimplantation development.

(A) The Spearman correlation coefficient (r1) between endogenous DNA methylation level of the promoter regions (green) and the relative expression levels of the corresponding RefSeq genes (red), the Spearman correlation coefficient (r2) between chromatin accessibility (purple) and the relative expression levels of the corresponding RefSeq genes (red), and Spearman correlation coefficient (r3) between the endogenous DNA methylation levels (green) and the chromatin accessibility (purple). The relative expression levels of the RefSeq genes were calculated as each gene's log_2(RPKM+1) divided by maximum of all genes' log_2(RPKM+1). The horizontal axis from left to right represents the expression levels from high to low. For calculation of the DNA methylation levels, 1kb upstream TSS and 500bp downstream TSS are used; and for calculation of the chromatin accessibility, 200bp upstream TSS and 100bp downstream TSS are used.

(B) Density plot of the relationship between endogenous DNA methylation variance and chromatin accessibility variance of the gene promoter regions. For calculation of the DNA methylation levels, 1kb upstream TSS and 500bp downstream TSS were used; and for calculation of the chromatin accessibility, 200bp upstream TSS and 100bp downstream TSS are used. Genes covered in more than five cells in each stage were analyzed.
