# Supplementary material for: Single-cell multi-omics sequencing of mouse early embryos and embryonic stem cells
Source: Cell Res. 2017 Jun 16;27(8):967–88. doi: 10.1038/cr.2017.82 (PMC5539349; doi:10.1038/cr.2017.82)
Supplement: Supplementary information, Figure S16 — The relationship between DNA methylation and chromatin accessibility during mouse preimplantation development. [file cr201782x16.pdf]

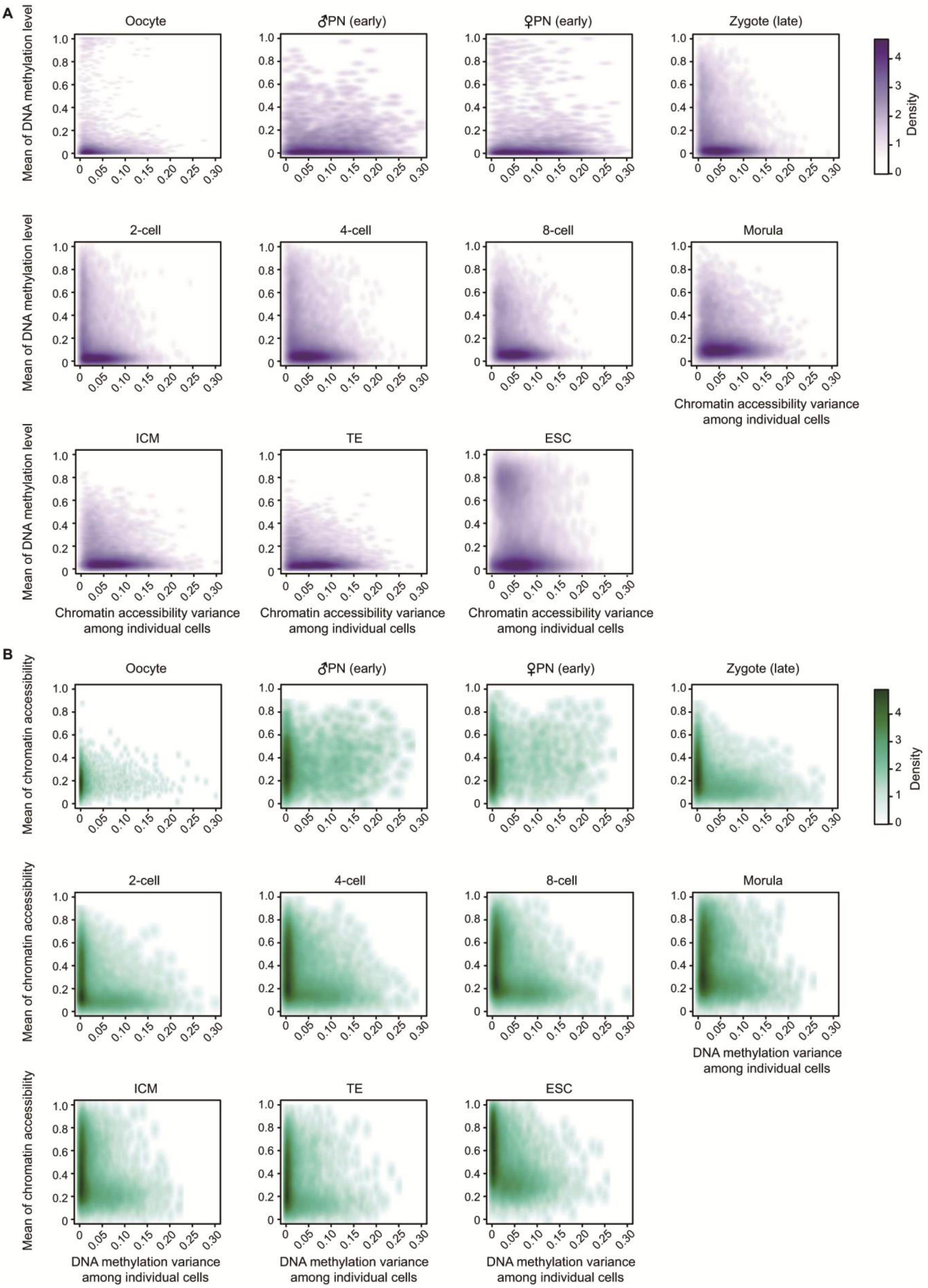
A
Oocyte
♂PN (early)
♀PN (early)
Zygote (late)
2-cell
4-cell
8-cell
Morula
ICM
TE
ESC
Mean of DNA methylation level
Chromatin accessibility variance among individual cells
Density
B
Oocyte
♂PN (early)
♀PN (early)
Zygote (late)
2-cell
4-cell
8-cell
Morula
ICM
TE
ESC
Mean of chromatin accessibility
DNA methylation variance among individual cells
Density

**Supplementary information, Figure S16.** The relationship between DNA methylation and chromatin accessibility during mouse preimplantation development.

**(A)** Density plot of the relationship between chromatin accessibility variance among individual cells at each developmental stage and the mean DNA methylation levels of the corresponding genes.

**(B)** Density plot of the relationship between DNA methylation variance among individual cells at each developmental stage and the mean chromatin accessibility levels of the corresponding genes.
