# Supplementary material for: Single-cell multi-omics sequencing of mouse early embryos and embryonic stem cells
Source: Cell Res. 2017 Jun 16;27(8):967–88. doi: 10.1038/cr.2017.82 (PMC5539349; doi:10.1038/cr.2017.82)
Supplement: Supplementary information, Figure S17 — Nucleosome positioning, ploidy and DNA replication timing of mouse early embryos. [file cr201782x17.pdf]

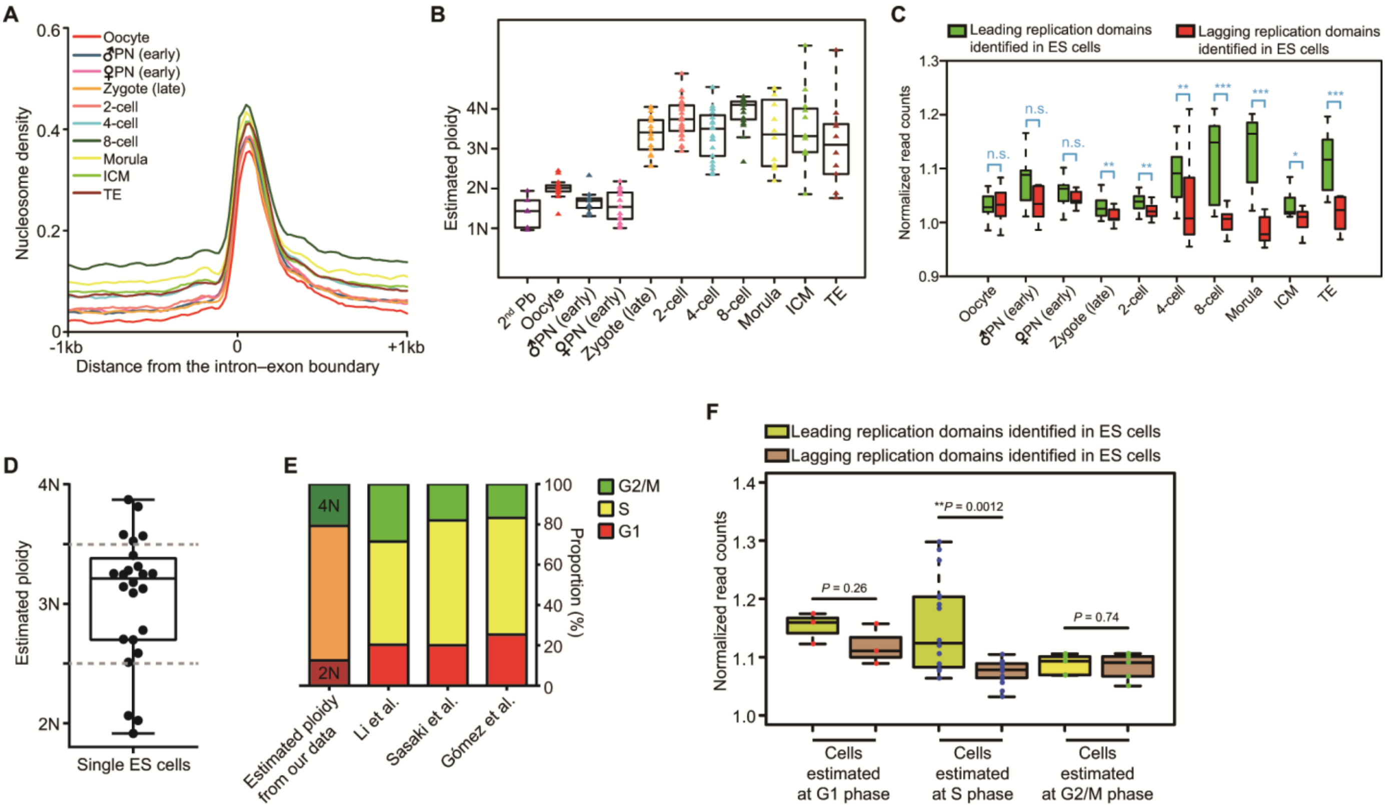

A
Oocyte
♂PN (early)
♀PN (early)
Zygote (late)
2-cell
4-cell
8-cell
Morula
ICM
TE
Nucleosome density
Distance from the intron–exon boundary
-1kb
0
+1kb
B
Estimated ploidy
1N
2N
3N
4N
2nd Pb
Oocyte
♂PN (early)
♀PN (early)
Zygote (late)
2-cell
4-cell
8-cell
Morula
ICM
TE
C
Leading replication domains identified in ES cells
Lagging replication domains identified in ES cells
Normalized read counts
n.s.
**
***
*
D
Estimated ploidy
Single ES cells
E
G2/M
S
G1
Proportion (%)
4N
2N
Estimated ploidy from our data
Li et al.
Sasaki et al.
Gómez et al.
F
Leading replication domains identified in ES cells
Lagging replication domains identified in ES cells
Normalized read counts
P = 0.26
**P = 0.0012
P = 0.74
Cells estimated at G1 phase
Cells estimated at S phase
Cells estimated at G2/M phase

Supplementary information, Figure S17. Nucleosome positioning, ploidy and DNA replication timing of mouse early embryos.

(A) Nucleosome density around the intron-exon boundaries across all the preimplantation stages.

(B) Boxplot of the deduced ploidy of each individual cell analyzed.

(C) DNA replication status in mouse early embryos. The published DNA replication domains of mouse ES cells were downloaded and used to analyze the DNA replication status in the mouse early embryos. The mean values of the counts in leading replication regions and in lagging regions were calculated for each single cell sample. A two-tailed *t*-test was used to calculate the statistical significance of copy number differences between the leading and lagging regions. * $P < 0.05$, ** $P < 0.01$, *** $P < 0.001$. ns denotes for not significant.

(D) Estimated ploidy of 24 individual ES cells.

(E) Deduced cell-cycle status of 24 individual ES cells and published cell-cycle status of mouse ES cells from 3 independent labs (Li *et al.*, *Proc Natl Acad Sci USA*, 2012; Rodriguez-Gomez *et al.*, *Am J Physiol Cell Physiol* 2012; Sasaki *et al.*, *Nat Med*, 2011).

(F) Boxplot of average read counts in DNA replication domains in each single ES cell. Three ES cells estimated as 2N in ploidy were assumed to be in G1 phase; sixteen ES cells estimated as between 2N-4N in ploidy were assumed to be in S phase, and the remaining five ES cells estimated as 4N in ploidy were assumed to be in G2/M phase. The average read counts of the leading replication domains were significantly higher than those of the lagging domains in S phase. A two-tailed *t*-test was used to calculate the *P*-value.

References

Li VC, Ballabeni A, Kirschner MW. Gap 1 phase length and mouse embryonic stem cell self-renewal. *Proc Natl Acad Sci USA* 2012; **109**:12550-12555.

Rodriguez-Gomez JA, Levitsky KL, Lopez-Barneo J. T-type Ca2+ channels in mouse embryonic stem cells: modulation during cell cycle and contribution to self-renewal. *Am J Physiol Cell Physiol* 2012; **302**:C494-504.

Sasaki M, Kawahara K, Nishio M, *et al.* Regulation of the MDM2-P53 pathway and tumor growth by PICT1 via nucleolar RPL11. *Nat Med* 2011; **17**:944-951.
