# Supplementary material for: Single-cell multi-omics sequencing of mouse early embryos and embryonic stem cells
Source: Cell Res. 2017 Jun 16;27(8):967–88. doi: 10.1038/cr.2017.82 (PMC5539349; doi:10.1038/cr.2017.82)
Supplement: Supplementary information, Figure S18 — Copy number variations in mouse preimplantation embryos. [file cr201782x18.pdf]

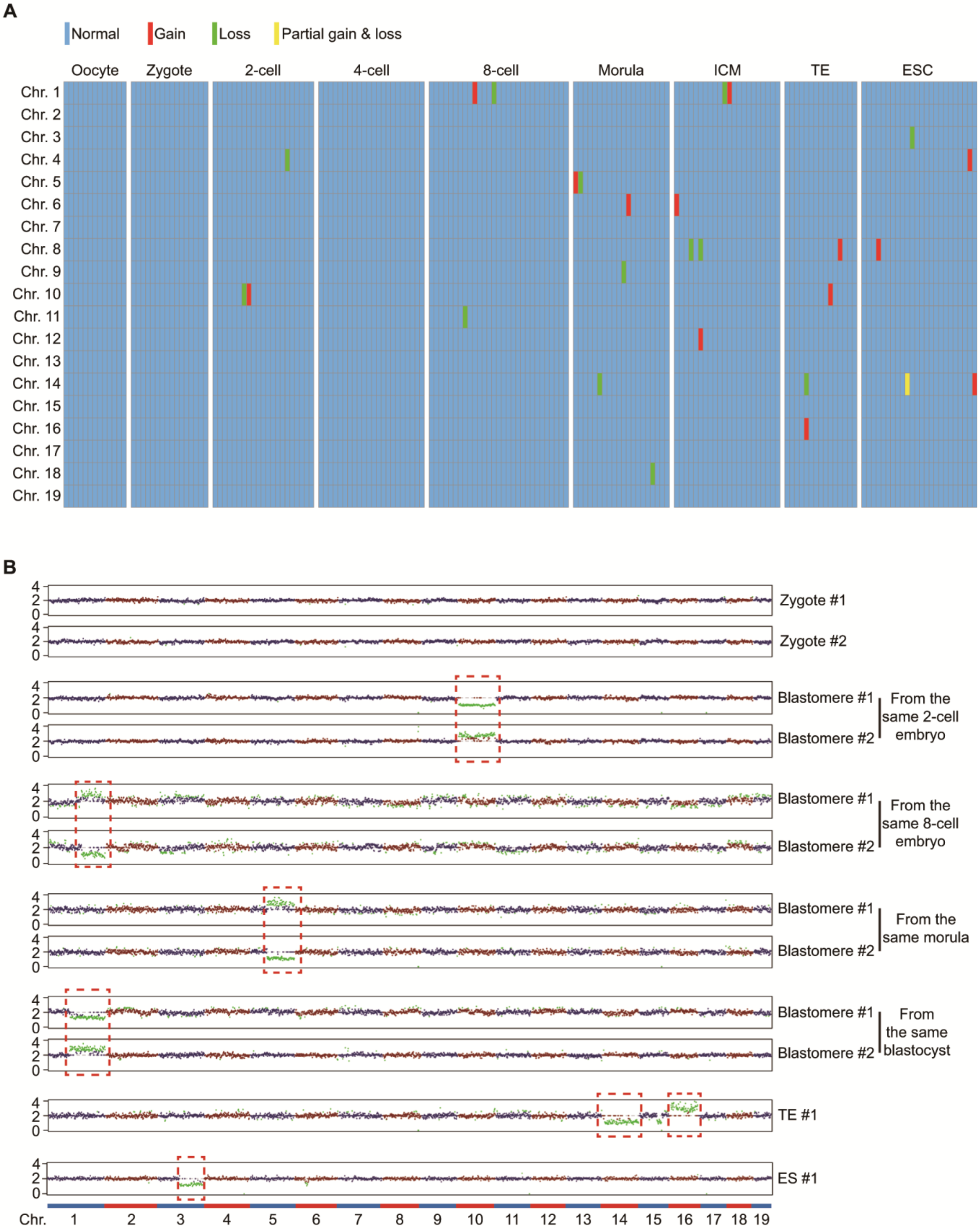
A
Normal
Gain
Loss
Partial gain & loss
Oocyte
Zygote
2-cell
4-cell
8-cell
Morula
ICM
TE
ESC
Chr. 1
Chr. 2
Chr. 3
Chr. 4
Chr. 5
Chr. 6
Chr. 7
Chr. 8
Chr. 9
Chr. 10
Chr. 11
Chr. 12
Chr. 13
Chr. 14
Chr. 15
Chr. 16
Chr. 17
Chr. 18
Chr. 19
B
Zygote #1
Zygote #2
Blastomere #1
Blastomere #2
From the same 2-cell embryo
Blastomere #1
Blastomere #2
From the same 8-cell embryo
Blastomere #1
Blastomere #2
From the same morula
Blastomere #1
Blastomere #2
From the same blastocyst
TE #1
ES #1
Chr.
1 2 3 4 5 6 7 8 9 10 11 12 13 14 15 16 17 18 19

Supplementary information, Figure S18. Copy number variations in mouse preimplantation embryos.

(A) Summary of blastomeres with CNVs during preimplantation development.

(B) Representative blastomeres with CNVs in mouse preimplantation embryos. Note that windows with normalized count either above 2.5 or below 1.5 were marked with green color.
