# Supplementary material for: Single-cell multi-omics sequencing of mouse early embryos and embryonic stem cells
Source: Cell Res. 2017 Jun 16;27(8):967–88. doi: 10.1038/cr.2017.82 (PMC5539349; doi:10.1038/cr.2017.82)
Supplement: Supplementary information, Data S1 — Single-cell COOL-seq Protocol [file cr201782x22.pdf]

Supplementary information, Data S1.

Single-cell COOL-seq Protocol

Artical: Single-cell multi-omics sequencing of mouse early embryos and embryonic stem cells

Authors: Fan Guo*, Lin Li*, Jingyun Li*, Xinglong Wu, Boqiang Hu, Ping Zhu, Lu Wen, Fuchou Tang
Affiliations: Beijing Advanced Innovation Center for Genomics, Ministry of Education Key Laboratory of Cell Proliferation and Differentiation, College of Life Sciences, Peking University, Beijing 100871, China; Biomedical Institute for Pioneering Investigation via Convergence, Peking University, Beijing 100871, China; Peking-Tsinghua Center for Life Sciences, Peking University, Beijing 100871, China; Group of Translational Medicine, Department of Obstetrics and Gynecology, Ministry of Education Key Laboratory of Obstetric, Gynecologic & Pediatric Diseases and Birth Defects, West China Second University Hospital, Sichuan University, Chengdu, Sichuan 610041, China; Academy for Advanced Interdisciplinary Studies, Peking University, Beijing 100871, China
*These authors contributed equally to this work
Correspondence: guofan@scu.edu.cn (F.G.), tangfuchou@pku.edu.cn (F.T.)

Day 1

In vitro methylation of single-cell nuclei and release of genomic DNA

1. Lyse a single cell in 3.5 µl of lysis buffer (50mM Tris-HCl (pH 7.4), 50mM NaCl, 10mM dithiothreitol, 0.25mM EDTA, 0.25mM phenylmethylsulfonyl fluoride and 0.5% NP-40, plus 1pg λDNA), keep on ice for 10-15 min.
2. Add the GpC methyltransferase M.CviPI and S-adenosylmethionine (New England Biolabs) to the lysate.
    - *In vitro* methylation mix (5 µl):
        - 3.5 µl of lysate
        - 0.15 µl of 10 × M.CviPI buffer
        - 1.25 µl of M.CviPI (4 U/ µl)
        - 0.1 µl of SAM (8mM)
        - **5 µl in total**
3. Incubate the mixture in a thermocycler at 37 ℃ for 30 min and then at 65 ℃ for 25 min.
4. After *in vitro* methylation, add 0.5 µl of 20 mg/ml protease (QIAGEN) and incubate for another 3 hours at 50 ℃, 20 min at 75 ℃.

Bisulfite conversion of single-cell genomic DNA

1. Use the MethylCode Bisulfite Conversion Kit (Invitrogen; Catalog no. MECOV-50).
2. Single-cell genomic DNA is bisulfite converted at 98 ℃ for 10 min, 64 ℃ for 2.5 hrs and then 4 ℃ for storage.
3. Genomic DNA is eluted with 20 µl of elution buffer (EB; QIAGEN).

Random priming of bisulfite converted gDNA

1. Prepare the first-round random priming mix:
    - 19.5 µl bisulfite-converted DNA

- 2.5 µl 10 × NEB buffer 2
- 1.0 µl of 10 µM scBS-seq-biotin-P5-N9-oligo1
- 1.0 µl dNTP (10mM each)
- **24 µl Total reaction**

2. Incubate at 65 ℃ for 3 min; 4 ℃ for pause.
3. Add 1 µl Klenow exo^{-} (50 U/µl, NEB), then 4 ℃ for 5 min, +1 ℃/15 sec to 37 ℃, 37 ℃ for 30 min.
4. After priming, incubate at 95 ℃ for 1 min.
5. Add 2.5 µl random priming mixture to the reaction, incubate at 4 ℃ for 5 min, +1 ℃/15 sec to 37 ℃, 37 ℃ for 30 min.
   - 1.0 µl of 25 pM scBS-seq-biotin-P5-N9-oligo1
   - 0.5 µl Klenow exo^{-} (50 U/µl, NEB)
   - 1.0 µl of 2.5 nM each dNTP
   - **2.5 µl in total**
6. Repeat the random priming , making it 6 rounds in total for a single cell.
7. Add 2 µl exonuclease I (20 U/µl, NEB), then incubate for 1 hr at 37 ℃. Then purify with AMPure XP Beads once, then elute in 50 µl EB buffer.

Day 2

Capturing biotinylated DNA on streptavidin beads

1. Take 20 µl of Dynabeads M280 Streptavidin beads into a 1.5-mL tube. Place the tube on the magnet stand to collect the beads.
2. Remove the supernatant, and add 50 µl of 2 × BW(Li) buffer to suspend the beads.
   - 2 × BW(Li) buffer (50ml):
   - 6.3g of LiCl
   - 0.5ml of 1M Tris-HCl (pH 7.4)
   - 0.1ml of 0.5M EDTA
   - add H_2O to 50ml total volume
3. Add the suspension of beads (in 50 µl of 2 × BW(Li) buffer) to the product obtained after AMPure XP Beads purification.
4. Incubate the tube at room temperature for 30 min.
5. Place the tube on the magnet stand to collect the beads, and then remove the supernatant.
6. Add 180 µL of 2 × BW(Li) buffer to the beads.
7. Place the tube on the magnet stand to collect the beads, and then remove the supernatant.
8. Suspend the beads in 180 µl of 0.1N NaOH solution, incubate at room temperature for 2 min.
9. Place the tube on the magnet stand to collect the beads, and then remove the supernatant.
10. Repeat steps 8 and 9 once again.
11. Add 180 µl of 2 × BW(Li) buffer to the beads.
12. Place the tube on the magnet stand to collect the beads, and then remove the supernatant.
13. Add 180 µl of EB buffer to the beads.
14. Place the tube on the magnet stand to collect the beads and remove the supernatant. Then suspend with 39 µl EB buffer.

Second-strand synthesis

1. Add the following 9 µl mix to the 39 µl random priming mixture, incubate the sample at 95 ℃ for 3 min.
   - 5 µl 10 × NEB buffer 2
   - 2 µl of 10 µM scBS-seq-P7-N9-oligo2
   - 2 µl dNTP (10 mM each)
   - **9 µl in total**
2. Then add 2 µl Klenow exo⁻ (50 U/µl, NEB), incubate at 4 ℃ for 5 min, +1 ℃/15 sec to 37 ℃, 37 ℃ for 90 min.
3. Wash the beads twice with 50 µl of EB buffer, and put the tube on the magnet stand to collect the beads and remove the supernatant.

PCR amplification and purification

1. Add the following mixture to the beads.
   - 1 µl of 15 µM Universal PCR Primer (NEB)
   - 1 µl of 15 µM PCR Index Primer (NEB, pre-indexed)
   - 25 µl of 2 × KAPA HiFi HotStart ReadyMix
   - 23 µl H_2O
   - **50 µl in total**
2. 95 ℃ for 3 min, 12 cycles of (98 ℃ for 20 sec, 65 ℃ for 30 sec, 72 ℃ for 1 min), 72 ℃ for 3 min and 4 ℃ hold.
3. Purified the DNAs with AMPure XP beads twice, elute with 30 µl EB finally.
4. Finally, libraries were pooled (quantified with qPCR) and sequenced on the Illumina HiSeq 2500 sequencer for 150-bp paired-end sequencing.
